# Counting on COVID-19 Vaccine: Insights into the Current Strategies, Progress and Future Challenges

**DOI:** 10.3390/biomedicines9111740

**Published:** 2021-11-22

**Authors:** Ramesh Kandimalla, Pratik Chakraborty, Jayalakshmi Vallamkondu, Anupama Chaudhary, Sonalinandini Samanta, P. Hemachandra Reddy, Vincenzo De Feo, Saikat Dewanjee

**Affiliations:** 1 Applied Biology, CSIR-Indian Institute of Technology, Uppal Road, Tarnaka, Hyderabad 500007, Telangana, India; 2 Department of Biochemistry, Kakatiya Medical College, Warangal 506007, Telangana, India; 3 Advanced Pharmacognosy Research Laboratory, Department of Pharmaceutical Technology, Jadavpur University, Kolkata 700032, West Bengal, India; pratik.chakraborty88@yahoo.com; 4 Department of Physics, National Institute of Technology, Warangal 506004, Telangana, India; vlakshmij@gmail.com; 5 Orinin-BioSystems, LE-52, Lotus Road 4, CHD City, Karnal 132001, Haryana, India; a.chaudhary-lifescience@outlook.com; 6 Department of Dermatology (Skin & Venereology), ESIC Medical College & Hospital, Patna 801103, Bihar, India; snspresi95@gmail.com; 7 Department of Internal Medicine, Texas Tech University Health Sciences Center, Lubbock, TX 79430, USA; hemachandra.reddy@ttuhsc.edu; 8 Department of Neuroscience & Pharmacology, Texas Tech University Health Sciences Center, Lubbock, TX 79430, USA; 9 Department of Neurology, School of Medicine, Texas Tech University Health Sciences Center, Lubbock, TX 79430, USA; 10 Public Health Department of Graduate School of Biomedical Sciences, Texas Tech University Health Sciences Center, Lubbock, TX 79430, USA; 11 Department of Speech, Language and Hearing Sciences, School Health Professions, Texas Tech University Health Sciences Center, Lubbock, TX 79430, USA; 12 Department of Pharmacy, University of Salerno, 84084 Fisciano, Italy

**Keywords:** coronavirus disease 2019 (COVID-19), SARS-CoV-2, vaccine, coronaviruses, reinfection, epidemiology, spike protein, ACE2 receptor, antigenicity, immunity

## Abstract

The emergence of a novel coronavirus viz., severe acute respiratory syndrome coronavirus 2 (SARS-CoV-2) in late 2019 and its subsequent substantial spread produced the coronavirus disease 2019 (COVID-19) pandemic worldwide. Given its unprecedented infectivity and pathogenicity, the COVID-19 pandemic had a devastating impact on human health, and its clinical management has been a great challenge, which has led to the development and speedy trials of several vaccine candidates against SARS-CoV-2 at an exceptional pace. As a result, several COVID-19 vaccines were made commercially available in the first half of 2021. Although several COVID-19 vaccines showed promising results, crucial insights into their epidemiology, protective mechanisms, and the propensities of reinfection are not largely reviewed. In the present report, we provided insights into the prospects of vaccination against COVID-19 and assessed diverse vaccination strategies including DNA, mRNA, protein subunits, vector-based, live attenuated, and inactivated whole/viral particle-based vaccines. Next, we reviewed major aspects of various available vaccines approved by the World Health Organization and by the local administrations to use against COVID-19. Moreover, we comprehensively assessed the success of these approved vaccines and also their untoward effects, including the possibility of reinfection. We also provided an update on the vaccines that are under development and could be promising candidates in the future. Conclusively, we provided insights into the COVID-19 vaccine epidemiology, their potency, and propensity for SARS-CoV-2 reinfection, while a careful review of their current status, strategies, success, and future challenges was also presented.

## 1. Introduction

Coronavirus disease 2019 (COVID-19) outbreak was first reported in Wuhan, China in December 2019, and was found to be caused by severe acute respiratory syndrome coronavirus 2 (SARS-CoV-2) which is a novel pleomorphic, positive-stranded RNA virus belonging to the Coronaviridae family. Quickly, it has become a global pandemic, infecting more than 176 million people and causing the death of more than 3.8 million individuals, that we are yet to recover from. Thus, an ongoing quest is being carried out for prophylaxis/therapy to prevent the transition from infection into serious forms of COVID-19 [1]. Though measures like physical distancing, use of masks, frequent sterilization, repurposing of existing drugs, etc. are being undertaken, the development of herd immunity through vaccination seems to be the most instrumental measure.

Many therapeutic strategies which can prove useful in the management of COVID-19 disease are underway, such as blocking the virus from binding cell receptors, preventing synthesis and replication of viral RNA, restoring innate immunity, modulating specific receptors/enzymes of the host, etc. [2,3,4,5]. However, amongst all such strategies to control the pandemic, the role of vaccines in preventing coronavirus (CoV) disease has been regarded as the most promising approach. The viral genome encodes several non-structural and structural proteins which include the spike (S), envelope (E), membrane (M), and nucleocapsid (N) proteins, which may play potentially instrumental roles to develop antigenic responses against the virus [6].

Scientists worldwide are in a race to develop safe and efficacious vaccine candidates against SARS-CoV-2 to curb the pandemic of COVID-19. Overwhelming attention has been paid to the S protein of the virus. The S protein makes up the studs outside the virus and is responsible for viral anchoring onto human cells through interaction with angiotensin-converting enzyme 2 (ACE2) receptors [1]. Hence, a vaccine expressing the S protein should induce a protective immune response without exposure to the whole virus in killed or attenuated form, i.e., the S protein itself is capable enough to act as the target antigen. Newer platforms use only the genetic material coding for S protein. Viral vectors with altered genetic payload (weakened viruses carrying sequences for the antigenic S protein) are also among the frontrunners in the race. Previously none of the adenovirus vectors, DNA vaccines, and mRNA vaccines had been approved by USFDA but the current pandemic has changed the trend. Other candidates in the race for vaccines include protein subunit vaccines and whole virus vaccines, some of which have displayed very promising results.

As of 28 October 2021, 49.10% of the global population has received at least one dose of COVID-19 vaccine, out of which 38.05% have been fully vaccinated [7]. Perturbations have been raised for the vaccines especially regarding their efficacy and the possibility of SARS-CoV-2 reinfection after being vaccinated. Mutation of an RNA virus is a matter of grave concern as it gives rise to newer strains, posing apprehensions that a vaccine developed for one strain might not be effective against a mutated strain. So far, several variants, such as B.1.1.7 (alpha, originated in the UK), B.1.351 (beta, originated in South Africa), P.1 (gamma, originated in Brazil), and B.1.617.2 (delta, originating from India) have been identified as major concerns [8,9]. These new mutants can spread faster, which raises the question of whether they may reduce the effectiveness of approved vaccines. World Health Organization (WHO) categorized variants C.37 (lambda, originated in Peru) and B.1.621 (mu, originated in Colombia) are of interest in this context [8]. Several other variants (B.1.466.2, B.1.525, B.1.526, B.1.617.1, B.1.619, B.1.620, B.1.630, B.1.1.318, C.36.3, R1, etc.) are still under monitoring [8]. In this review, we provided insights into the epidemiology, mechanism of action, and propensity for reinfection with CoV for the different vaccines administered worldwide.

## 2. Prospects of Vaccination against COVID-19

During the initial stages of SARS-CoV-2 infection, glycoprotein S of the virus (protruding out from the surface) binds with the ACE2 receptor of the host (human) cell. The S1 subunit interacts with the ACE2 receptor via the receptor-binding domain (RBD) while the S2 subunit is responsible for the fusion of the two cell membranes into a six-helix bundle core [10]. In the lower respiratory tract (LRT), viral S proteins lock themselves with epithelial cells of LRT including the alveolar epithelial cells-type 2. By utilizing the S2 subunit, the virus gains entry into lung cells. It seems that SARS-CoV-2 has some in-built mechanisms to withstand the antiviral activity of interferons. On spotting the viral antigens, the immune system kick-starts the production of antibodies against them. Further, cytokine and chemokine production is triggered by the antibodies, which in turn cause fluid build-up within the lungs. This fluid contains T-cells to kill viral particles. The CD8+ T cells, in combination with NK cells, destroy the virus-affected epithelial cells. With increasing accumulation of the fluid, lungs find it difficult to extract oxygen from air; as a result, dry cough (one of the preliminary symptoms of COVID-19 infection) starts as an attempt to exhale the fluids. Ninety-seven percent of patients exhibit symptoms like fever, dry cough, breathlessness, headache, joint pain, dizziness, diarrhea, nausea, etc. within 11.5 days of acquiring an infection, while the median incubation time for the virus is around 4–5 days [11]. Oestrogen can modulate the immune system to fight this infection more effectively, hence men are more likely to succumb to COVID-19 than women [12]. Clearly, the overdrive of the immune system in the form of cytokine storm leads to severe conditions amongst COVID-19 patients.

To cope with the uncontrolled replication of the virus, killer cells, e.g., monocytes and neutrophils are recruited, leading to overproduction of pro-inflammatory cytokines like IL-6, IL-8, IL-12, TNF-α, etc. (cytokine storm). Affected cells may present the viral antigens to CD8+ T cells while dendritic cells can present the antigens to CD4+ T cells. As a result of CD4+ T cell differentiation, memory T cells are produced to protect the human body from reinfection by similar strains. Plasma B cells also get into action by producing IgA, IgM, IgG, and other virus-specific antibodies. In a patient infected with COVID-19 for the second time, serum neutralizing antibodies were spotted within 8 days of hospitalization while IgM was absent [13].

Between SARS-CoV-2 and SARS-CoV, there are 27 amino acid replacements in the S protein whereas 102 and 61 amino acid substitutions are found in the NSP3 and NSP2, respectively [14]. To enter into the cell, SARS-CoV-2 binds with the human ACE2 receptor with a higher affinity than the SARS-CoV. MERS-CoV uses an entirely different receptor, dipeptidyl peptidase 4 [15]. As mentioned earlier, SARS-CoV-2 contains four structural proteins, namely, S, N, E, and M, and these are encoded by the 3′-end of the viral genome [16]. The S glycoprotein, a large multi-functional trans-membrane protein, plays a vital role in the attachment, fusion, and entry of the virus into the host cell. The S protein consists of S1 and S2 subunits. The S1 subunit has two functional domains viz. N-terminal domain and RBD. The S2 subunit has three operational domains, i.e., fusion peptide, heptad repeat 1, and heptad repeat 2. The stalk of the immune-dominant S protein is formed by the trimeric S2 subunit whereupon the S1 sits at the top. The high pathogenicity of the virus may be attributed to a furin cleavage site within the S protein. Conformational masking and glycan shielding has been hypothesized to let the S protein circumvent host immune cells [17].

Immunotherapy involving artificial triggering of the host immune system to elicit an immune response has been considered as an effective strategy for prophylaxis against infectious diseases [18]. Development of a vaccine that elicits the production of neutralizing antibodies to S protein is the primary aim of the researchers amongst the various COVID-19 vaccines (in use and trial). Many full-length genomes of SARS-CoV-2, isolated from various countries are now available for delineating polymorphisms in S protein and other important proteins with regard to vaccine development [19]. Epidemiological studies conducted in China have estimated that the reproduction number (R_0_) and growth rate of COVID-19 is very high [20]. Developing herd immunity against COVID-19 seems to be the only way out to get rid of the ongoing pandemic. However, the infection of more than 60% of the global population to generate this herd immunity poses an immense risk of fatality and other losses. Hence, constituting an effective vaccine is crucial and considered the only practical way to establish herd immunity. Several government and non-government agencies have come up with funding for vaccine development against COVID-19. To develop a safe and effective vaccine, it is critical that before every/any vaccine is released for public use, all pre-clinical and clinical trials are conducted with vigilance to avoid severe adverse effects. Before the advent of CoV, the fastest developed vaccine was the mumps vaccine which took about 5 years to make the cut. Within more or less a year of release of the genome sequence of SARS-CoV-2 in January 2020, multiple vaccines have been approved for public use worldwide. Clearly, uncontrolled haste could possibly worsen the situation. Fast-tracking vaccine development by combining various phases involves trials being conducted on smaller groups. Cooperation among various international organizations is critical at this point regarding the emergence of unwanted effects in various demographic groups, especially for the newer platforms being used. Exorbitant vigilance must be continued to prevent public use of any suspicious candidate vaccine under any kind of pressure. SARS-CoV-2 is an RNA virus, generally having a high mutation rate. Genetic instability has long been considered to represent a challenge to develop long-lasting protection against RNA viruses due to the emergence of newer and resistant variants with time [21]. The protective effect of a candidate vaccine is highly attributed to antibodies against the S protein, against the RBD of the S protein to be more precise. Often, healed patients display high titers of SARS-CoV-2 neutralizing antibodies [22]. Effective vaccination may not only significantly reduce the occurrence and severity of illness but also prevent disease spread.

## 3. Strategies to Develop Vaccines against COVID-19

Since the initial years of the current century, CoVs were thought to cause mild flu-like symptoms. Serial outbreaks of SARS in 2002, MERS in 2012, and COVID-19 from 2019 onwards have demonstrated their pathogenicity globally. Global losses of human resources and economy due to the ongoing COVID-19 pandemic make vaccines highly desirable, as presently no definite drug is available against CoV. Vaccine research targeting SARS-CoV-2 worldwide is exploring various strategies in search of a safe and effective vaccine. The various candidates identified in the search undertaken so far include inactivated virus vaccines, recombinant viral vaccines, subunit vaccines, nucleic acid vaccines, and attenuated vaccines (Table 1). Nucleic acid vaccines are relatively newer candidates in the process of vaccine development. Of note, DNA vaccines need to reach the nucleolus of the cell, while RNA vaccines can act from within the cytoplasm [23]. They express desirable antigens inside the cell to induce an immune response.

Nanotechnology can play a vital role in vaccine development as both nanoparticles and viruses operate at similar size scales. The ability of nanoparticles to enter cells, to enable expression of nucleic acids, and/or directly target immune cells for co-delivery of antigens and adjuvants make them attractive platforms for vaccine design.

### 3.1. DNA Vaccine

In 1986, the US patent was granted for a cDNA-based vaccine against the canine CoV [24]. DNA vaccines are attractive candidates owing to their simplicity, stability, and ease of production. The S, M, and N proteins of CoV have been reported to induce cellular and humoral responses making them essential components to be included for various DNA vaccine candidates. In a mouse model experiment conducted in 2004, DNA vaccine-associated expression of S protein generated both T-cell and neutralizing antibody responses, besides lowering replication of SARS-CoV in the respiratory tract [25].

DNA vaccines can deliver CoV genes to the human cells mostly through recombinant plasmid DNA. The plasmid contains a mammalian promoter along with a transgene sequence encoding the desired antigen, S protein in this case. The principle relies on DNA translocation within the nucleus of the cell. In the nucleus, using the host cell machinery antigenic proteins are expressed by the delivered viral genetic material. Thus, they are much safer than a live attenuated virus or inactivated virus regarding chances of pathogenicity. Moreover, these vaccines use human cells as production houses to increase the antigenic load, thus decreasing individual dose requirements. Antigen-presenting cells can be transfected directly with the delivered genetic material. Expressed antigens are loaded onto MHC I and MHC II molecules due to the cross priming potential [26]. The antigens are either released by exosomes or apoptotic bodies which lead to recognition by antigen presenting cells, and further evolution of humoral or cytotoxic immune responses. However, a low risk of viral mutation still coexists by integration of transfected DNA with host somatic DNA, leading to irregularities in genetic expressions.

Devices like electroporators and gene guns can be used to improve the delivery and uptake of the genetic material by antigen-presenting cells. Antigen-presenting cells will, in turn, present the antigens to naive CD4+ and CD8+ T cells of the secondary lymphatic system, thereby generating cellular immune responses. Further, antigens produced later in the body will also reach secondary lymph organs and activate B cells to produce antibodies. Suitable adjuvants may also be co-administered to boost adaptive immunity.

### 3.2. mRNA Vaccine

This approach is arguably the most rapid and flexible amongst different vaccination strategies being taken up worldwide. To combat a rapidly spreading virus, using mRNA instead of protein is a faster approach as a vaccination strategy. The mRNA vaccines deliver a short viral mRNA sequence to express the antigenic protein inside the host cell. Generally, local innate immune responses are induced by mRNA vaccines, which lead to potent adaptive immunity [27]. Since these vaccines are not involved in genetic recombination, there stands no chance of dysregulation in host genetic expressions, unlike the DNA vaccines [27]. The immunogenicity of these mRNA vaccines is comparable to that of the inactivated viruses, while at the same time these are devoid of the risks of accidental virulence associated with whole viral vaccines [27]. They are also superior to protein subunit vaccines as they do not pose the risk of protein contamination, besides bypassing the time consuming and laborious steps of purification and standardization of viral proteins. However, the requirement of very low temperatures (≤−20 °C) for long-term storage of mRNA vaccines makes them difficult to use at the community scale.

Generation of RNA vaccines involves reactions between a DNA plasmid template and a recombinant RNA polymerase [28]. A sequence analogous to capping and a poly(A) tail is added at 5′ and 3′ ends, respectively, to form a mature and stable RNA sequence. The hydrophilic nature and strongly negative charge of mRNA pose difficulty in cellular uptake of mRNA in vivo. Further, naked mRNA is highly susceptible to ribonuclease in the cytoplasm. Thus, efficient carriers, preferably lipidic nanoparticles, are required to pack the mRNA in a stable injectable form [27]. Self -amplifying mRNA vaccines contain both the genes encoding desired antigen and the genes required for self-replicating enzymes. The main difference between these two types of RNA vaccines lies in the time taken for the onset of action. The conventional one presents with prompt antigen production within the host cell and therefore humoral and cellular immune responses are induced quickly. On the other hand, while delayed antigenic production is seen with the self-amplifying ones, these produce higher yield and confer equivalent protection in the long run with a much lower dose. RNA, being the minimally immunogenic genetic vector, circumvents the risk of anti-vector immunity to a large extent even on repeated administrations. The mRNA vaccines also have the capability to induce both antibody production and T-cell responses, since the protein antigen i.e., the S protein in most cases is produced using host machinery [29,30]. However, antigenic expression after mRNA vaccination is transient, limiting its persistence in the human body and thus calling for repeated administrations at certain intervals.

### 3.3. Protein Subunit Vaccine

Subunit vaccines are made up of either synthetic peptides or recombinant proteins responsible for eliciting immunogenicity. Since these vaccines consist of only certain immunogenic fragments instead of the whole viruses, they are quite efficacious as well as safer. Further, small defined peptide fragments are easy to scale up and pose fewer risks of side effects. As already mentioned, a number of structural proteins, such as S, E, M, and N, expressed by SARS-CoV-2, may act as antigens to activate the immune response. Generally, either booster doses or co-administration of suitable adjuvants is required along with peptide subunits to elicit an immunogenic response to the desired level. Currently, the highest number of vaccine candidates against SARS-CoV-2 is using this strategy [31].

Amongst the various structural proteins expressed by SARS-CoV-2, S proteins are the most suitable candidates as vaccines since they contain sites for both binding of the receptor (S1) and membrane fusion (S2). Subunit vaccines based on S protein are expected to generate antibodies preventing the binding of virus and later fusion of membranes thus conferring double-layer protection against entry of the virus. Interestingly, the infectivity of SARS-CoV-2 is counteracted by human mAb that binds to S protein through N-terminal, thereby preventing binding of the receptor through syncytia formation. On the contrary, few epitopes of S protein of SARS-CoV-2 can elicit potentially harmful immune responses. Hence, it is a must to identify and eliminate such epitopes beforehand. The S protein has been reported to protect mice and monkeys from SARS-CoV challenge by inducing serum neutralizing antibodies [32]. The S protein is also shown to be responsible for eliciting responses by CD4+ and CD8+ T-cells [33]. The RBD is a 193-amino acid segment in the center of the S1 subunit responsible for binding to receptors present on the target cells. It has been reported that vaccines based on RBD can produce antibodies in mice and rabbits displaying 50% neutralizing titers at >1:10,000 serum dilution [34]. The RBDs are advantageous over complete S protein in that, unlike S protein, they produce only neutralizing antibodies owing to the absence of immunodominant regions responsible for non-neutralizing antibody formation. However, the absence of some potentially important epitopes may also make them inferior to S protein as far as an immunogenic response is concerned [35]. Recombinant sequences comprising peptides from different variants can be used to protect against multiple viral strains. Counteracting antibodies have also been produced using M proteins which are generally responsible for the proper configuration of the viral envelope [36]. N proteins have demonstrated eminent T cell response and, when used in conjunction with adjuvants, have also produced IgG antibodies, but these antibodies fail to protect from subsequent infection [37]. As a general rule, prefusion-stabilized viral glycoproteins are usually more immunogenic, thus being more attractive vaccine targets. The delivered antigen must maintain its surface chemistry and profile of the original pre-fusion S protein to preserve the epitopes for inducing proper antibody responses [38]. Further, using receptor binding motifs as antigens would enhance the neutralizing antibody response.

### 3.4. Recombinant Viral Vector Vaccine

Recombinant viral vector vaccines consist of a less virulent, actively dividing virus recombined with a gene of interest encoding desired antigenic protein. Replication of this recombinant virus inside the host cell would produce CoV proteins leading to activation of the immune system. Viral vectors are selected in such a way that they can infect human cells easily and can be detected by antigen-presenting cells. To minimize chances of pre-existing host immunity, non-human viruses or rare serotype viruses are usually selected as vectors [39]. Accidental integration of viral genome with host genome may lead to uncontrolled replication resulting in disaster. In some cases, the viral vector proteins may also serve as adjuvants to elicit an immune response against the highly pathogenic antigen [40]. Genetically engineered viral vectors expressing SARS-CoV-2 proteins have been at the center of attraction right from the start of the COVID-19 vaccine development. Increasing interests have been focused on this type as vector-based vaccines can be constructed relatively fast and used even without an adjuvant. The S protein, specifically the RBD of S-protein has been identified as a neutralizing epitope which could be actively pursued for the development of viral vector-based vaccines [41]. Replication-competent vectors are required at lower-dose to elicit strong responses as the multiplying vectors can result in enhanced antigen presentation whereas replication-defective vectors need to be administered in higher doses.

To serve as a viral vector, adenovirus comes up with advantages like ease of administration along with non-pathogenicity to human beings. Back in 2003, an adenovirus vector containing genes for N, S, and M proteins of SARS-CoV successfully induced antibody response to S1 fragment and T cell response to N protein in *Rhesus macaques* monkeys [42]. Poxvirus is another potential vector candidate owing to greater insert size, cytoplasmic gene expression, and long-lasting immune response in the human. Newcastle disease virus could have been another possible vector candidate, but a higher risk of pathogenesis and probable carcinogenicity is holding it back in the race.

### 3.5. Live Attenuated Vaccine

Historically, live attenuated vaccines have been most successful against intracellular pathogens [43]. Attenuated live virus mimics natural infection to elicit a long-lasting immune response but with a lower risk of pathogenicity. Generally, though this type of vaccine is highly efficacious, safety is a paramount concern. Live attenuated vaccines present the entire viral antigens to the host immune system, deliver antigens to respective cell compartments, and are easily presented through antigen-presenting cells similar to natural infection, thus generating cytotoxic T cell responses, antigen-specific effectors, and also memory cells. Therefore, this type of vaccine can generate T-cell responses as well as antibody responses with long-lasting immune memory. The most critical step during their development is the optimization of balance between high immunogenicity and low virulence. Reversal of virulence inside the host becomes a concern in a few cases. In the case of CoV, deletion of genes encoding E protein has successfully developed non-virulent mutants [44]. Hamsters vaccinated with the mutant under discussion have raised the level of serum neutralizing antibodies and protected from clinical symptoms and replication of SARS-CoV in the respiratory tract [45]. Some other mutations are also under research. Due to the large genome size of CoV, it takes a longer time to prepare a non-virulent clone. Reversal to virulent form may effectively be prevented through gene replacement knockout strategy more effectively than chemical treatment or irradiation [46]. For attenuation, exhaustive long-term repetitive cultures are required. Generally, via repeated replication in the host, wild-type pathogens are allowed to accumulate mutations that adapt to the new host, progressively lowering the virulence to humans. Even after that, the attenuated strain might revert to the wild genotype. CoVs are known to frequently recombine in nature, further complicating the development of the live attenuated vaccines. These types of vaccines are therefore generally not recommended for immuno-compromised individuals considering the risks.

### 3.6. Whole Killed Vaccine/ Inactivated Virus Vaccine

A whole killed virus vaccine refers to a virus devoid of its ability to infect and replicate within a host cell but retaining its immunogenicity. Generally, the whole virus is neutralized by heat, radiation, or chemical treatment thus requiring considerably fewer efforts than live attenuated vaccines. Inactivated viral vaccines present the same epitopes to the immune system as natural infection by the virus rather than only the S protein in the case of some other technologies discussed earlier. Formaldehyde and β-propionolactone are the most popular chemical agents used for inactivation. Successful sterilization of SARS-CoV, in bulk, has been demonstrated by ultraviolet radiation [47]. Inactivated SARS-CoV has been observed to induce neutralizing antibodies in mice models in the past [48]. Generally, administration of booster dose and co-administration of adjuvants help to generate better, long-lasting adaptive cellular immunity responses. Since dead pathogens are administered, this traditional vaccine technology is comparatively safer for immune-compromised patients.

### 3.7. Virus-Like Particles

Virus-like particles are composed of only the capsid layer without any infectious nucleic acid. They effectively present different surface antigens and/or multiple copies of one antigen mimicking virus structures thereby invoking immune responses [49]. The clustering of antigenic epitopes provides scopes of cognate activation of B cells and enhances antibody response [50]. The safety profile is almost unquestionable since they are devoid of any genetic material.

### 3.8. Oral Mucosal Vaccine

**Figure 1:**
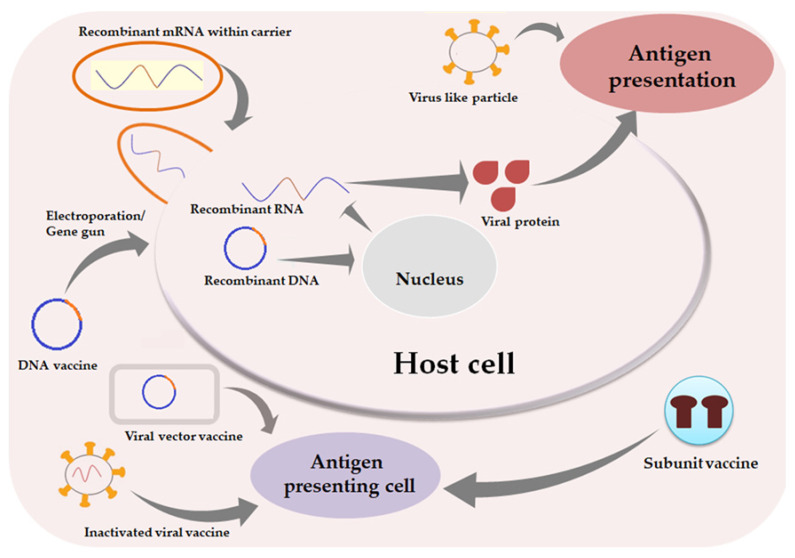
Strategies used in the development of common SARS-CoV-2 vaccine candidates.

All aforementioned vaccines were intended for parenteral use. However, the high abundance of ACE2 receptors in the mucosal linings of the digestive tracts (especially in ileum and colon) supports the development of the idea of an oral mucosal vaccine against SARS-CoV-2 [51]. A couple of oral mucosal anti-COVID-19 vaccines are in the pipeline, which are mainly recombinant viral vector vaccines designed for oral delivery. Vaxart formulated a recombinant COVID-19 vaccine tablet for oral delivery, which is now in phase I trial (NCT04563702) [52]. This vaccine contains adenovirus vector encoding genes for S and N proteins of the virus. OraPro-COVID-19™, another oral vaccine is underway by a UK-based company iosBio in collaboration with BioCell Corporation, Auckland, New Zealand. It uses non-replicating adenovirus-5 (Adv5) expressing S glycoprotein [51]. Both the vaccines are capable of achieving both humoral and cellular immune responses through intestinal lymphoid tissues [51]. Considering the abundance of ACE2 receptors in the mucosal linings of enterocytes, these vaccines were formulated as thermally-stable enteric coated formulations to protect the vaccine components from the acidic environment of the stomach [51]. Figure 1 schematically depicts the common vaccine strategies.

## 4. Vaccines Approved for Public Use

**Figure 2:**
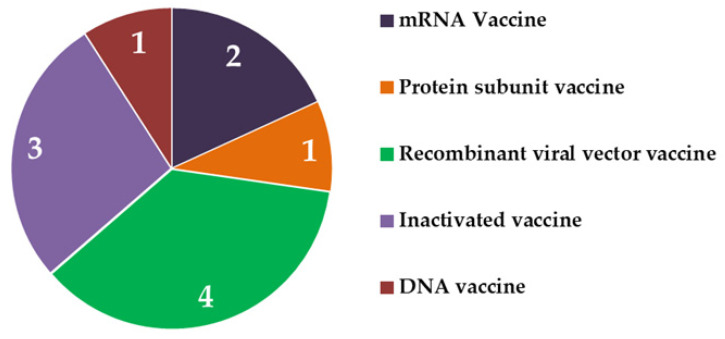
Distribution of approved vaccines based on vaccine types. Recombinant viral vector vaccines dominate the list followed by inactivated vaccines, mRNA vaccines, protein subunit vaccine and DNA vaccine.

Within a year of the emergence of novel CoV, vaccines are being deployed in countries, giving faith to our ability to fight the COVID-19 pandemic. The goal of developing an effective vaccine against CoV has fetched huge investments from multiple governments and non-government agencies across the globe. During Phase I, small groups of people receive the candidates under clinical trial. In Phase II, the scope is expanded, and vaccine candidates are administered to people who have characteristics (such as age and physical health) similar to those for whom the new vaccine is intended. In Phase III, the vaccines are given to thousands of people in a multicentric approach and tested for their efficacy and safety. After approval, post-marketing surveillance continues as the fourth phase. Regulatory authorities continue to ensure safety through regular/periodic monitoring. Currently, we are on the verge of a very critical step of immunization of the majority of the global population through approved vaccine products. To date, all the approved vaccines belong to either of the five types i.e., mRNA vaccine, viral vector vaccine, protein subunit vaccine, inactivated virus, and DNA vaccine (Figure 2).

### 4.1. Vaccines Approved by WHO for Global Application

WHO, in collaboration with other agencies, is aiming to work with vaccine manufacturers to offer low-cost vaccines to countries under the COVAX initiative. So far, WHO has approved six vaccines against COVID-19 (Table 2).

#### 4.1.1. Pfizer-BioNTech (mRNA Vaccine)

This is the first COVID-19 vaccine granted by WHO on 31 December 2020. This is an mRNA-based vaccine developed collaboratively by three pharmaceutical companies of Germany, USA, and China. The vaccine BNT162b2 is composed of nucleoside-modified mRNA (4284 nucleotides long sequence) encoding a mutated form of the full-length S protein of SARS-CoV-2 delivered in the form of lipid nanoparticles encapsulating the nucleic acid. The vaccine is a suspension for intramuscular injection administered as a series of two doses (0.3 mL each) 21 days apart each dose consisting of 30 μg mRNA vaccine embedded in lipid nanoparticles. Comirnaty in the USA and Pfizer-BioNTech COVID-19 vaccine in Europe contain the same formulation, and thus can be used interchangeably. The mRNA BNT162 codes for the RBD of S protein of SARS-CoV-2 besides including T4 fibritin-derived trimerization domain to elicit an immune response. The RNA sequence consists of a 5′ cap, a 48-base signal peptide, and two proline substitutions, K986P and V987P, allowing the spike to adopt a prefusion-stabilized conformation reducing the membrane fusion ability, increasing expression, and stimulating neutralizing antibodies. The 2P proline substitutions in the S protein were originally developed at the University of Texas at Austin, TX, USA for a vaccine against camel–flu, predominant in the Middle East. WHO recommends a two-dose schedule of BNT162b2 three to four weeks apart. The vaccine BNT162b2 (Comirnaty) has demonstrated 95% efficacy against symptomatic SARS-CoV-2 infection [53]. In some countries, BNT162b2 is recommended for minors (≥12 years) also. Lustig and colleagues [54] highlighted the requirement of the timely administration of the second dose, particularly in the elderly and immunosuppressed population. An Italian survey on anti-SARS-CoV-2 IgA response in baseline seropositive and seronegative individuals receiving BNT162b2 suggested the possibility of considering delaying/dropping the second dose of the vaccine in baseline seropositive individuals [55]. However, further detailed study is required to conclude anything regarding this concern. In a press release dated 8 July 2021, Pfizer-BioNTech has ignited the possibility of a third booster dose of the vaccine [56]. In line with most of the pre-existing vaccines, the efficacy of BNT162b2 has been found to decrease with the increasing age of the recipient [57,58].

#### 4.1.2. Astrazeneca/University of Oxford (Viral Vector Vaccine)

The vaccine prepared by Oxford-AstraZeneca is available in the market as Covishield or Vaxzevria. In February 2021, WHO recommended the use of this vaccine for all adults. It is currently approved in more than 130 countries. It is a monovalent vaccine that consists of a chimpanzee adenovirus DNA vector i.e., a recombinant and replication-deficient (ChAdOx1) vector, and encodes the S glycoprotein of SARS-CoV-2 [59,60]. The non-replicating viral vector vaccine is currently designated as AZD1222. L-Histidine analogs are also present in the formulation. The vaccine expresses SARS-CoV-2 S immunogen in a trimeric pre-fusion conformation; to stabilize the expressed S-protein in the pre-fusion conformation the code sequence was not modified in the pre-fusion conformation [57,60]. Two separate doses of 0.5 mL each are provided in the ChAdOx1nCoV-19 vaccination course. After the first dose, the second dose should be given after 4–12 weeks [61]. Persons who receive the first dose of this vaccine should receive the second vaccine dose to complete the course of vaccination. Each dose of this vaccine consists of more than 2.5 × 10^8^ infectious units of ChAdOx1-S [61]. The vaccine is given in the form of suspension for injection through intramuscular injection. According to a review of findings on the dosing interval of the ChAdOx1nCoV-19 vaccine, it has been found that the most important factor for vaccine efficacy is the dosing interval, and not the dosing level [62]. This is consistent with previous research which supports greater effectiveness over a longer period of time in other vaccines such as those for influenza and Ebola [62]. After a gap of 12 or more weeks between the first and second dose, the study found that vaccine efficacy reached 82.4% (95% CI 62.7% to 91.7%). The efficacy was only 54.9% when the two doses were given less than six weeks apart [63].

According to a report published in British Medical Journal, after a study of 2000 healthy and young volunteer workers, the rollout of Oxford-AstraZeneca COVID-19 vaccine in South Africa was stopped, reporting that it did not protect from mild and moderate disease owing to the new variant (501Y.V2) that emerged there [64]. In a cohort study in the UK, both BNT162b2 and ChAdOx1nCoV-19 have been found to demonstrate comparable efficacies against the B.1.1.7 variant of SARS-CoV-2 [65].

#### 4.1.3. Johnson and Johnson (Viral Vector Vaccine)

Janssen is a non-replicating viral vector vaccine (Ad26.COV2.S) to fight the menace of COVID-19. Ad26.COV2.S is a recombinant, replication-incompetent adenovirus serotype 26 vector encoding a full-length, stabilized S protein of SARS-CoV-2 WA1/2020 strain. It uses the same systems AdVac and PER.C6 earlier successfully used for developing the Ebola vaccine by the same sponsor company. The vaccine is recommended as a single intramuscular injection of 0.5 mL to adults delivering 5 × 10^10^ viral particles. Due to insufficient data on vaccine co-administration, a minimum gap of 14 days is recommended with any other kind of vaccination for other disorders. According to an update by WHO on 25 June 2021, Ad26.COV2.S is safe and effective at protecting people from extremely serious risks of COVID-19, including death, hospitalization, and severe disease. 28 days after inoculation Ad26.CoV2.S displayed an efficacy of 85.4% against severe disease and 93.1% against hospitalization [66]. A single dose of Ad26.COV2.S demonstrated the efficacy of 66.9% against symptomatic moderate and severe SARS-CoV-2 infection in clinical trials [66]. In search of design elements for CoV S protein, replacing Ad26 vector encoding a membrane-bound stabilized S protein with a wild-type signal peptide (Ad26.COV2.S) elicited potent neutralizing humoral immunity and cellular immunity that was polarized towards Th1 IFN-γ in mice [67]. In a comparative study by Mukhopadhyay and colleagues, Ad26.COV2.S ranked second to the rapid elicitation of immunogenicity and protective efficiency in non-human primates among six vaccine candidates based on data available until October 2020 [68]. During preclinical developments, Ad26.COV2.S was found to protect *Rhesus macaques* monkeys from SARS-CoV-2 after single immunization [69]. Recently, a G614 spike SARS-CoV-2 virus variant Syrian hamster model has proven the success of Ad26.COV2.S in preventing COVID-19 and associated LRT infections [70]. A second dose of the vaccine to Syrian hamsters was found to be beneficial for the G614 spike SARS-CoV-2 variant with optimum immunogenicity without vaccine-associated enhanced respiratory diseases. Low dose Ad26.COV2.S has been demonstrated to impart protection of SARS-CoV-2 in *Rhesus macaques* [71].

#### 4.1.4. Moderna (mRNA Vaccine)

This vaccine is an mRNA vaccine (mRNA-1273). The vaccine comprises lipid nanoparticle-encapsulated, nucleoside-modified mRNA encoding the stabilized prefusion S glycoprotein S-2P of SARS-CoV-2 [72]. The mRNA encodes S protein in such a way that when the vaccine is injected, immune cells process the mRNA, and the subsequent proteins would be marked for destruction [73]. The dosage regimen includes two doses of 0.5 mL (100 μg lipid nanoparticle encapsulated mRNA) each to be administered through the intramuscular route 28–42 days apart. The vaccine is about 94.1% effective against COVID-19, starting 14 days after the first dose [74]. Storage at −20 °C is recommended for the long term; however, after thawing it is stable at cold conditions for up to 30 days thus making it very suitable for widespread community use [75]. Corbett et al. noticed robust SARS-Co-2 neutralizing activity with mRNA-1273 in non-human primates without any pathogenic change in the respiratory system [76]. It has been observed that mRNA-1273 induces both potent neutralizing antibodies and CD8+ T cell responses for protection against SARS-CoV-2 infection in the lungs and noses of mice without imparting any immunopathological manifestation [77]. In addition to that, a high pseudovirus neutralizing antibody response was noticed in mice expressing a mutated form of the S protein, D614G [77].

#### 4.1.5. Sinopharm (Inactivated Virus Vaccine)

This is an inactivated novel CoV (19-nCoV-CDC-Tan-HBO2 strain, optimal replication, highest virus yield in Vero cell) vaccine (BBIBP-CorV/verocell) developed in China against SARS-CoV-2 to stimulate the immune system. β-propionolactone bonded with the genes of virus in such a manner that the virus cannot replicate, but its S proteins remain intact. Two doses (3–4 weeks apart) of 0.5 mL (6.5 U inactivated SARS-CoV-2 antigen + 0.225 mg aluminium hydroxide adjuvant) through intramuscular route have been recommended by WHO. Vaccine efficacy was found to be 79% against both symptomatic COVID-19 infection (14th day onwards after second dose) and COVID-19-associated hospitalization [78]. Phase III clinical trial data (NCT04984408) are insufficient to determine vaccine efficacy against persons with comorbidities [79]. It can be stored under cold temperature conditions making it suitable for widespread community use. Sinopharm, CoronaVac, and Covaxin use similar technologies to prepare inactivated virus vaccines against COVID-19 [80]. During animal experiments, 2 doses (2 μg each) of BBIBP-CorV successfully induced high titer values of neutralizing antibodies against SARS-CoV-2 in mice, rats, guinea pigs, rabbits, Cynomolgus monkeys, and Rhesus monkeys [81].

#### 4.1.6. Sinovac Biotech (Inactivated Virus Vaccine)

CoronaVac (formerly PiCoVacc) is an inactivated virus (formalin treated) vaccine using alum as an adjuvant, developed by Sinovac Biotech, Beijing, China. CoronaVac is recommended in 2 doses of 0.5 mL (600 SU SARS-CoV-2 antigen) each intramuscular injection 2–4 weeks apart. CoronaVac has been claimed to be 51% effective against symptomatic COVID-19 infection and 100% effective against severe COVID-19 infection and hospitalization, according to a phase III clinical trial in Brazil, from day 14 onwards after the second dose [82]. In a phase I/II study in China involving 144 and 600 participants, respectively, in phase I and phase II between 16 April 2020 and 5 May 2020, 3 μg dose had been recommended for further trials based on safety, immunogenicity, and production capacity [83]. Pain at the injection site is the most reported adverse effect post-vaccination. Though it is recommended for the adult population, only limited safety data for individuals ≥60 years is currently available. WHO granted an emergency use listing for CoronaVac on 1 June 2021.

### 4.2. Vaccines Approved Regionally

Many countries have approved one or more vaccines based on outcomes. Some have been approved by WHO later. Still, some vaccines are there, which have succeeded to satisfy the regulatory bodies of a few countries but are yet to obtain global acceptance.

#### 4.2.1. Sputnik V (Viral Vector Vaccine)

Sputnik V (Formerly, Gam-COVID-Vac) is a recombinant adenovirus vaccine rAd26 and rAd5 developed in Russia. This adenovirus vaccine is the World’s first registered combination vector vaccine against COVID-19, the ‘V’ standing for ‘Victory’ over COVID-19. Based on phase I and phase II clinical trial data only, it was approved for use in Russia in August 2020. According to an interim analysis published later, the efficacy of Sputnik V is 91.6% [84]. It is to be administered through intramuscular injection in two doses, first dose rAd26 and after 21 days, second dose rAd5. Novel CoV gene encoding S protein is integrated with viral vector DNA. Thus, unmodified full-length S protein generates an antigenic response in the host. A cold chain of subzero temperature is not required for storage of the lyophilized powder which paves the way for easier community use. Sputnik Light is a single dose of rAd26 to be used as a third booster dose if needed after at least six months. Adenovirus 26 and adenovirus 5 are used as vectors for the expression of SARS-CoV-2 S protein. The heterologous recombinant adenovirus approach with two varying serotypes aims to overcome any pre-existing adenovirus immunity [85]. Initially, Sputnik V has faced lots of criticism for unseemly haste with low transparency, but the outcome and interim analysis reports published in the Lancet have attempted to do away with the allegations of non-transparency [84,86,87,88,89,90]. The Gamaleya Research Institute (Moscow, Russia) claimed that Sputnik V is more than 90% effective against the B.1.617.2 variant of CoV [91].

#### 4.2.2. EpiVacCorona (Protein Subunit Vaccine)

EpivacVacCorona is a peptide vaccine developed in Russia. The vaccine comprises three synthetic peptides mimicking viral S protein. These peptides are conjugated to a carrier protein, a fusion product of viral nucleocapsid protein, and a bacterial maltose-binding site protein. The viral portion of the chimeric protein is responsible for immunization, aluminium hydroxide serves as an adjuvant. It is to be injected in two doses 21 to 28 days apart through the intramuscular route. Currently, it is approved for emergency use in Russia, Belarus, and Turkmenistan. Immunogenicity and protectivity of the peptide candidate were assessed in a preclinical study [92]. EpiVacCorona was administered in two doses (260 μg each) 14 days apart to hamsters, ferrets, African green monkeys, and *Rhesus macaques* monkeys. The vaccine was 100% successful to generate virus-specific antibodies in animals. In hamsters, dose-dependent immunogenicity was observed along with prevention from pneumonia, in ferrets, EpiVacCorona speeded up clearance of CoV from the upper respiratory tract (URT); COVID-associated pneumonia was prevented in non-human primates. Two clinical trials (NCT04780035 and NCT04527575) aiming to assess the tolerability, safety, immunogenicity, prevention efficacy, and reactogenicity of EpiVacCorona comprising of 3000 and 100 volunteers, respectively, are yet to post the results [93,94]. Currently, the vaccine has been approved in Russia, Turkmenistan, and Belarus.

#### 4.2.3. Bharat Biotech (Inactivated Virus Vaccine)

BBV152 (Covaxin) is a completely ineffective SARS-CoV-2 viral particle that contains the RNA surrounded by a protein shell, but the genetic material is chemically modified so that it cannot replicate [95]. The vaccine consists of one of the two different adjuvants and a single inactivated whole SARS-CoV-2 virion. The adjuvant is either an aluminum hydroxide gel (Algel) or a novel TLR7/8 agonist (imidazoquinolinone) adsorbed Algel [96]. A separate T-helper-cell 1 (Th1) antibody response with increased levels of SARS-CoV-2-specific IFN-γ and CD4 cells was further induced by the formulation containing the TLR7/8 agonist [96]. The dosing regimen of Covaxin consists of two doses (6 μg whole virion inactivated antigen of NIV 2020-770 strain + adjuvant in each dose) given at a 28 days interval [95]. Components of Covaxin include BBV152A, BBV152B, and BBV152C. In a study, immunogenicity of this inactivated virus vaccine formulated with both the adjuvants was determined in rabbits, mice, and rats using three concentrations (3, 6, and 9 μg) [96]. The results show that BBV152 formulations produce significantly high antigen binding and neutralizing antibody titers at concentrations of 3 and 6 μg in all three species irrespective of adjuvants. Moreover, the vaccine maintains excellent safety profiles at 6 μg [96].

#### 4.2.4. Cansino Biologics (Viral Vector Vaccine)

Convidicea (Ad5-nCoV), also known as PakVac is a recombinant vaccine against COVID-19 using adenovirus type 5 vector encoding SARS-CoV-2 S protein. Ad5-nCoV is based on replication-defective adenovirus type 5 as the vector to express the S protein of SARS-CoV-2. The vaccine has displayed an efficacy of 65.7% against moderate symptoms of COVID-19 and 91% efficacy to protect from severe disease [97]. Comfortable storage conditions of 2-8oC and single dosing requirements make it a potentially popular vaccine candidate. During preclinical studies, Ad5-nCOV single dose was found to protect BALB/c mice completely from URT and LRT infection by mouse-adapted SARS-CoV-2 and to protect ferrets from URT infection by SARS-CoV-2 wild variant [98]. A single-dose regimen comprising of 0.5 mL intramuscular solution (≥4 × 10^10^ viral particles) makes it a more convenient option than multidose alternatives. Currently, it is authorized for emergency use in 10 countries.

#### 4.2.5. Zydus Cadila (Plasmid-DNA Vaccine)

The DNA plasmid-based COVID-19 vaccine, ZyCoV-D is the world’s first DNA vaccine to get the regulatory nod for use. It has been authorized for emergency use in India by CDSCO on 20 August 2021 for people aged ≥12 years. This is also the first needle-free vaccine (Tropis Pharma-jet-based delivery platform) approved globally. In an indigenous collaborative venture by Cadila Healthcare and Biotechnology Industry Research Assistance Council, India, the vaccine comprises a plasmid vector carrying genetic material encoding the S protein of SARS-CoV-2 to interfere with viral entry through membrane protein. The recombinant plasmid acts as a vector to carry the genetic material coding for S1 protein of CoV into the cell to generate an immune response [99]. The plasmid enters the host cell nucleus as an episome without integrating into host DNA. Intradermal administration of three doses (0.2 mL each) is recommended on day 0, day 28, and day 56.

Plasmid DNA comes with the inherent advantage of elimination of possibility of vector-based immunity. On interim analysis of phase III trial data, Cadila Healthcare (Ahmedabad, India) announced the efficacy of ZyCoV-D to be 66.6% against symptomatic COVID-19 and 100.0% against moderate to severe disease [100]. Manufacturers have also claimed that the vaccine is useful for individuals >12 years, making it an option for adolescents of India. The plasmid constructs have been transformed into *Escherichia coli* to boost production [101]. The immunogenic potential has been evaluated in mice, guinea pigs, and rabbits at different doses intradermally, while preclinical toxicology was studied in rats and rabbits [101]. ZyCoV-D, in preclinical studies induced neutralizing antibody response along with Th-1 response and elevated interferon-γ levels. Yadav et al. evaluated the immunogenicity and protective efficacy of ZyCoV-D formulations at varying doses in *Rhesus macaques* [102]. The vaccine at 2 mg dose successfully induced S1 specific IgG and neutralizing antibody titers, which increased gradually on the viral challenge for up to 2 weeks protecting from lung disease. Evaluation of nasal swab, throat swab, and bronchoalveolar fluid also confirmed viral clearance. Enhanced proliferation of lymphocytes and production of IL-5 and IL-6 were also evidenced. The signal peptide produced from plasmid genetic material includes RBD for ACE2 receptor to hinder viral entry into the host cell during future infections [103].

### 4.3. Success of Approved Vaccines

In a clinical trial consisting of 43,448 volunteers (≥16 years), BNT162b2 was given in two doses (30 μg each) 21 days apart [104]. Out of 21,720 vaccine recipients, 8 presented with the onset of COVID-19 after 7 or more days of the second dose, while 1 from the vaccine group presented with severe COVID-19 infection after the first dose. Overall, BNT162b2 was 95% effective in preventing COVID-19 (95% CI, 90.3 to 97.6). To be precise, vaccine efficacy of 52.4% and 94.8% was observed after a single dose and at least 7 days after 2 doses, respectively. Emerging evidence revealed that BNT162b2 can efficiently neutralize both N501 and Y501 variants (having mutated RBDs) of SARS-CoV-2 [105,106]. In an observational study in Israel among healthcare workers from December 2020 to January 2021, vaccine efficacy of 89–91% was reported after 15–28 days of the first dose [107]. Adjusted rate reductions of COVID-19 disease were 47% (95% CI 17–66) and 85% (71–92) for days 1–14 and days 15–28 after the first dose, respectively. These early reductions of COVID-19 rates provide support for delaying the administration of the second dose in countries facing vaccine shortages [107]. According to a real-world dataset assessment, inoculation with BNT162b2 reduced viral load substantially between 12–37 days of the first dose, indicating the lower grade of infection and lesser possibility of virus spread after vaccination [108]. Another study on this concern revealed single-dose vaccination to be protective starting from day 14 onwards and reaching a peak at day 21, though scientists are yet to accept the interpretation unanimously and some interpret the same results in a not-so-encouraging conclusion [109]. Persons previously infected with COVID-19 develop stronger T and B cell-mediated immune responses after a single dose of BNT162b2 than unaffected individuals [57]. Higher titers of neutralizing antibodies have also been observed in vitro in those individuals compared to the unaffected ones after a single-dose of BNT162b2 [55]. A two-dose schedule of BNT162b2 could successfully neutralize engineered variants of SARS-CoV-2 with mutations in S protein (69/70 deletion) and E484K [110]. Although vaccination yielded more or less similar neutralizing antibody titers for the engineered mutated variants compared to the parental strains, these kinds of deliberate engineering situations may prove highly dangerous. BNT162b2 has been found to remain effective against a SARS-CoV-2 pseudovirus bearing mutation in B.1.1.7 S protein [111]. Among the 40 sera tested, neutralization was only slightly decreased against the B.1.1.7 lineage pseudovirus, more evidently in individuals aged below 55 years. A cohort study by Kustin and colleagues [112] suggests reduced efficacy of BNT162b2 against B.1.1.7 and B.1.351 variants of SARS-CoV-2. In an attempt to evaluate the effectiveness of a three-dose schedule for hemodialysis patients [113] it was observed that 93% of the native patients yielded an antibody titer > 50 UA/mL after 2 doses while its value increased to 93% after the 3rd dose. Among the 10 non-responders after the second dose, 2 responded significantly after the 3rd dose (17 to 568 UA/mL and 35 to 923 UA/mL); 3 patients demonstrated no change in antibody titers after the 3rd dose, 2 demonstrated a significant decrease, while 5 denied taking 3rd dose. Morales-Núñez and peers [114] reported the production of neutralizing antibodies in 100% cases in both naive and previously infected individuals after 2 doses with tolerable adverse events and the possibility of immune-senescence. Results from a real-life study in Italy discovered the emergence of IgG directed towards the S protein of SARS-CoV-2 in 99.88% of the vaccinated population (1765 healthcare workers) 3 weeks after the second dose of BNT126b2 [115]. Younger recipients and those with a previous history of COVID-19 infection came up with better immunogenic responses than others. In a population of patients suffering from mast cell disorders (*n* = 26), BNT162b2 vaccine was well-tolerated without any serious adverse events despite the generally increased possibility of anaphylaxis [116]. No significant increase in serum STL level was observed after either of the doses nor was any clinical symptom of mast cell mediator release observed. Given the acute vaccine shortage worldwide, Ramos and colleagues [117] investigated the antibody response after a single dose of BNT162b2 to examine the probable benefits of fast administration of the vaccine to a large population to protect from COVID-19. Findings suggest that before the second dose inoculation, 95.3% already had anti-SARS-CoV-2 IgG, half of them even had antibody concentrations against RBD of the virus; however further studies are needed to conclude on a mass scale. From a retrospective cohort study, no increased risk was apprehended regarding vaccination for patients of inflammatory bowel disorder being treated with immunosuppressive agents, vaccine effectiveness in such patients was found to be highly comparable with that in the reference population [118]. Pottegård and colleagues reported lower incidences of thrombohemorrhagic adverse events with BNT162b2 than with ChAdOx1nCoV-19 [119]. Ram and colleagues [120] examined the effect of BNT162b2 on patients undergoing immune cell therapy. Sixty-six patients after allogeneic HCT treatment and fourteen patients after CD-19 based CART therapy were vaccinated with BNT162b2. Impressive immunogenicity was observed; 57% of individuals after CART infusion and 75% after allogeneic HCT demonstrated humoral and/or cellular immune response on vaccination; 12% after first dose and 10% after second dose developed cytopenia, while three developed GVHD exacerbation after each dose. One graft rejection, later on, was thought to be related to vaccination. Recent pieces of evidence suggest BNT162b2 to be effective against newer variants of CoV such as B.1.526, B.1.1.7, B.1.429, and B.1.617.2 [121,122,123].

Lower immunogenicity of BNT162b2 has been reported in liver transplant patients than regular individuals while the results in liver transplant cases remain superior to other organ transplant cases [124]. Haemodialysis patients above 60 years of age have demonstrated lower antibody responses than healthy individuals [125]. Strengert and colleagues reported variable humoral and cellular immune responses on vaccination with BNT162b2 in hemodialysis patients [126]. In such patients, both anti-SARS-CoV-2 IgG and neutralization efficacy were reduced compared to healthy individuals. T-cell mediated IFN-γ release after stimulation with SARS-CoV-2 S peptides was also reduced. In Lithuania, patients with hematological malignancy depicted lower median anti-S1 IgG responses after 2 doses of vaccination than healthy individuals [127]. Among the patient population, patients actively treated with Bruton-tyrosine kinase inhibitors, ruxolitinib, venetoclax, and anti-CD20 antibody therapy displayed poorer antibody response than the untreated patients. Elderly patients with myeloma demonstrated a lower neutralizing antibody response against SARS-CoV-2 than healthy individuals [128]. Mucosal sites represent the primary entrance route for SARS-CoV-2 to the human body. Anti S protein IgG and IgA total antibody titer and presence of neutralizing antibodies were assessed in serum and saliva of 60 healthcare workers after two weeks of both first and second doses of BNT162b2 [129]. From the results, it was evident that BNT162b2 can trigger neutralizing antibodies in serum, but not in saliva. Thus, this vaccine may not be very useful to stop the spreading of the virus from one human to another, however, is likely to protect vaccinated individuals via the systemic immune response.

Results of interim efficacy for two of the four ongoing studies with ChAdOx1nCoV-19 from the UK and Brazil in 11636 participants between 18–55 years are available [130]. The ChAdOx1nCoV-19 recipients were not admitted with COVID-19-related complications, while 10 hospital admissions (2 of them serious) took place from control teams [130]. The vaccine efficacy, after primary dose analysis (combining dose groups), was 70.4% (95.8% CI 54.8–80.6%) [130]. A number of 30 out of the 5807 participants in ChAdOx1 nCoV-19 Group, and of 101 (1.7%) out of the 5829-control group were found to be infected with COVID-19, over 14 days after the second dose. A study was undertaken in Denmark following the reports of thromboembolism associated with ChAdOx1nCoV-19 vaccine with 4,915,426 individuals of age group 18–99 (follow up time 38,449,703 person-years) and 3,963,153 individuals of age group 18–64 (follow up time 29,537,310 person-years) [131]. In a test-negative case-control study in the UK, the national immunization management system successfully linked 156,930 adults aged 70 years and older, which reported COVID-19 symptoms between 8 December 2020 and 19 February 2021, with their data on vaccines [132]. Participants who were vaccinated with AZD1222 had underlying risks of COVID-19 infections compared to non-vaccinated people in the first 9 days. The occurrence of ChAdOx1-S (S protein) was observed in blood between 14 to 20 days after vaccination [113]. The efficacy of the vaccine against COVID-19 infection was found to be 60% (41% to 73%) after 28 to 34 days of vaccination, and from day 35 onwards, efficacy shot up to 73% (27% to 90%) [113]. The need for hospital admission reduced by 37% (3% to 59%) after receiving the single dose of the vaccine [132,133]. An updated report of 17178 participants, among whom 9696 (56.4%) are female, 12975 (75%) are white and 14,413 (83.9%) are aged between 18–55 years, 1792 (90%) are aged between 56–69 years and 973 (5.7%) are aged 70 years or older, has been analyzed by [134,135,136]. Participants were divided into 2 groups, one receiving two standard doses and the other group receiving a low dose followed by a standard dose. Results show an overall vaccine efficacy of 66.7% (95% CI 57.4–74.0%) more than 14 days after the second dose of the vaccine against the appearance of COVID-19 symptoms. In the patients who received two standard doses, the efficacy of the vaccine was 63.1%, and it was 80.7% in those who received a low dose along with the standard dose. The effect of vaccines after a standard single dose was particularly high in exploratory analyses at 76.0% between day 22 and day 90, with a minimum decrease in antibody levels during that time [137]. The vaccine efficacy was significantly higher at 81.3% after two standard dose intervals given at 12 weeks, compared to 55.1% at less than 6-week intervals, supporting longer interval immunization strategy. These findings were supported by immune-supportive studies in participants under 55 years of age that showed anti-SARS-CoV-2 IgG spike two-fold higher responses in people with a dosing interval of 12 weeks compared to less than six weeks having a geometrical mean ratio of 2.32 (95% CI 2.01–2.68) [63,137]. In a randomized controlled trial with healthcare workers for detecting IgG spike after vaccination, it was found that 864 out of 890 (97.1%) were seropositive after 14 days of vaccination with ChAdOx1nCoV-19 [138]. Previously infected healthcare workers are more likely to be seropositive. All 470 healthcare workers tested after the second dose of vaccination were found to be seropositive. Quantitative antibody response in recipients of ChAdOx1nCoV-19 with infection and without infection were 10,095 (5354–17,096) and 435 (203–962) AU/mL, respectively [138]. A Phase III interim analysis in Peru, Chile, and the US found that the Oxford University and AstraZeneca-produced vaccine was 79% effective in preventing COVID-19 symptoms and 100% effective in preventing severe diseases and hospital admission [139]. In the trial, 141 symptomatic cases of COVID-19 were reported among 32,449 participants, with 2:1 randomized to the vaccine group and placebo group. Four weeks apart, they were given both doses of the vaccine [139].

A phase I-IIa trial was conducted with Ad26.COV2.S on 805 participants in two age groups (18–55 years and ≥65 years) [140]. Neutralizing antibody titer against wild-type of CoV was detected (geometric mean titer value 212–354) in 90% or more of all participants on day 29 after the first dose. The antibody titers remained stable for at least 71 days besides a 2.6–2.9-fold increase after the second dose. Spike binding antibodies also followed the trend of neutralizing antibodies. On day 15, CD4+ T-cell responses were detected in 76 to 83% of the participants receiving two doses and in 60 to 67% of those receiving a single dose, with a clear bias toward type 1 helper T cells. The CD8+ T-cell responses were slightly lower in single-dose recipients compared to double-dose recipients. To evaluate the immunogenicity of Ad26.COV2.S, a trial on 25 participants within 22–52 years was conducted [141]. Binding and neutralizing antibodies emerged rapidly by day 8 after initial immunization in 90% and 25% of vaccine recipients, respectively. By day 57, binding and neutralizing antibodies were detected in 100% of vaccine recipients after a single injection. On day 71, the geometric mean titer of spike-specific binding antibodies was 2432 to 5729, and the geometric mean titer of neutralizing antibodies was 242 to 449 in the vaccinated groups. A variety of antibody subclasses, Fc receptor binding properties, and antiviral functions were induced along with CD4+ and CD8+ T-cell responses at the interim endpoint of day 71. Ad26.COV2.S elicited both humoral and cellular immune responses cross-reacting with B.1.351 variant, thus protecting *Rhesus macaques* monkeys from B.1.351 challenge [142]. Ad26.COV2.S induced lower binding and neutralizing antibodies against B.1.351 than against WA1/2020, but elicited CD8 and CD4 T cell responses comparable to those against WA1/2020, B.1.351, B.1.1.7, P.1, and CAL.20C. Sera from the recipients of a single dose of Ad26.COV2.S demonstrated susceptibility to Ad26.COV2.S-induced serum neutralization for B.1.351 and P.1 variants that contain similar mutations in the RBD, while much lower efficacy as observed for B.1.617.2 (delta) variant [143]. In South Africa, D614G and 501Y.V2 strains of CoV demonstrated high levels of cross-reactivity in spike-binding assay (*n* = 120) at least 29 days post-vaccination [144]. Interestingly, in a population subset (*n* = 27), 82% of the tested sera showed no detectable neutralizing antibody against 501Y.V2. Thus, it has been suggested that even low levels of neutralizing antibodies can contribute to protection from moderate to severe disease in combination with roles played by Fc effector function and T cells in protection against 501Y.V2 variant.

In a phase-I study on ≥56 years old persons, 100 μg dose of mRNA-1273 induced higher binding and neutralizing antibody titer than lower doses [145]. In another phase I study on 45 healthy participants between 18–55 years old, mRNA-1273 induced anti-SARS-CoV-2 immune responses in all participants without any identifiable trial-limiting safety concerns [146]. During the Phase III clinical trial of the mRNA-1273 vaccine, 30,420 participants were enrolled within the age group of 18–95 years [74,147]. Among the participants, 7000 were aged over 65 years, and 5000 were below 65 populations presenting with high-risk chronic diseases. Around 63% of the trial subjects were white along with 6000 Hispanics and more than 3000 Blacks. On 2 months follow-up, findings indicated 94.1% (95% CI = 89.3–96.8%) vaccine efficacy in persons without previous history of SARS-CoV-2 infection. Severe COVID-19 infection occurred in 30 participants (all placebo recipients) with one recorded death [74]. Among the subjects, 10 instances of hospitalization were documented for COVID-19 infection among which 9 were from the placebo group, further supporting vaccine-mediated protection from COVID-19 complications. More than 86% efficacy has been observed across age, sex, race, and in subjects with underlying medical conditions. In both the vaccine and placebo groups, the frequency of serious adverse events was very low. In GRADE (Grading of recommendations, assessment, development and evaluations) evidence assessment, the vaccine displayed high certainty regarding protection from symptomatic SARS-CoV-2 infection, moderate certainty in preventing COVID-associated hospitalization, and very low certainty regarding protection from asymptomatic SARS-CoV-2 infection [72]. Tré-Hardy and colleagues, in an interim analysis of vaccination surveillance, concluded that anti-SARS-CoV-2 antibodies persisted for up to 3 months after vaccination onset; however, a significant decrease in antibody levels was observed in some seronegative cases even earlier than that. Hence, the third dose of vaccination may seem to be a reality in future [148]. Mustafa and peers attempted to administer graded doses of the vaccine to administer the second dose to patients reporting immediate hypersensitivity reactions after the first dose [149]. However, both of the subjects showed negative results when skin prick tests was performed following the recommendation of Banerji et al. [150]. One of them temporarily developed pruritis after administration of the vaccine, which vanished without any medication. After 3–4 weeks, none of them had any adverse reactions and both of them had developed IgG directed against the S protein of SARS-CoV-2, indicating a successful vaccination. Despite a decrease in titers of binding and neutralizing antibodies in the human with time, mRNA-1273 has been proven to impart humoral immunity responses for at least 119 days [151]. Krammer and colleagues observed that a single dose of mRNA-1273 elicited rapid immune responses in seropositive persons, with post-vaccination antibody titers similar to or more than that in seronegative persons receiving two doses [152].

Xia and colleagues conducted phase I (96 participants, 18–59 years) and phase II (224 participants, 18–59 years) clinical trials with BBIBP-CoR-V between 12 April 2020 and 27 July 2020, in Henan province, China [153]. Participants receiving three doses through 56 days in the phase I trial displayed only a little difference in neutralizing antibody titer values with similar CIs compared to participants receiving two doses in phase II. In phase II again, participants receiving two doses 28 days apart showed higher neutralizing antibody titers than those receiving two doses 14 days apart. Beijing Institute of Biological Products conducted phase I and phase II clinical trials with 192 volunteers between 18–80 years and 448 adult volunteers between 18–59 years, respectively, at Shangqiu City Liangyuan District Center for Disease Control and Prevention in Henan province, China [110,154]. BBIBP-Cor-V was able to elicit antibodies against SARS-CoV-2 in all trial subjects on day 42 after the last dose. Two doses (4 μg each) 21–28 days apart produced more neutralizing antibody titers than a single dose (8 μg) or two doses (4 μg each) 14 days apart. UAE had claimed 86% efficacy for the vaccine in December 2020 based on the phase III trial while within a few days Sinopharm claimed it to be 79% based on phase I and phase II data [155]. A Wuhan-based phase III trial has claimed vaccine efficacy to be 72.5% [156]. Phase III trials have also taken place in Peru, Egypt, Argentina, Bahrain, Jordan, and Pakistan [156,157]. In a small-scale trial, 12 volunteers received BBIBP-CorV [126]. Based on serum neutralizing titer values, the vaccine was proven to provide immunity against two variants of SARS-CoV-2 viz. 501Y.V2 and D614G (wild type). Geometric mean titer values were 110.9 (95% CI 76.7–160.2) and 71.5 (51.1–100.1) for D614G and 501Y.V2, respectively. In a cross-sectional survey in Jordan, 38.2% of the 2213 respondents received BBIBP-CorV and no life-threatening post-vaccination side effect was recorded [158]. According to Huang and colleagues, BBIBP-Cor-V is effective against the South African variant of CoV [159]. A Srilanka-based study concluded that BBIBP-Cor-V generates antibody responses comparable to natural infection against B.1.351 and B.1.617.2 variants of CoV [160].

In a randomized, double-blind, placebo-controlled phase I/II trial with CoronaVac in Hebei, China on people aged ≥60 years, 72 persons were enrolled for phase I between 22 May 2020 and 1 June 2020, and 350 persons were enrolled for phase II between 12 June 2020 and 15 June 2020 [161]. In phase I, seroconversion after the second dose was observed in 100% of subjects in the 3 μg group, and 95.7% of participants in the 6 μg group. In phase II, seroconversion was observed in 90.7% in 1.5 μg group, 98.0% in the 3 μg group, and 99.0% in the 6 μg group. Results also indicated optimum neutralized antibody titer with a 3 μg dose. In a study based in Nanjing, China, sera of 93 recipients of 2 doses were collected and assayed for neutralization activity against seven variants of SARS-CoV-2 [162]. After 14 days of second dose, 82% of serum samples could neutralize wild-type pseudovirus. Coronavac also proved effective against D614G, B.1.1.7, and B.1.429 variants. In a test-negative study in Brazil, a single-dose of CoronaVac was evidenced as more effective against symptomatic infection by the gamma variant of CoV than the two-dose regimen [163]. Another test negative, case-control study was performed in Brazil from 17 January 2021 to 29 April 2021 on a population aged ≥70 years [164]. CoronaVac was found to be 42% effective against transmission of P.1 variant of CoV after 2 doses. A phase I/II clinical trial was undertaken with CoronaVac at Hebei, China on a juvenile population aged 3–17 years in late 2020 [165]. Phase I included 72 participants in 3 age groups and 2 dosage regimes while phase II included 480 participants and 2 dosage regimens. Adverse events were mostly mild to moderate pain at the injection site (13%). Humoral immune response was triggered by vaccination, and results of neutralizing antibody titers supported the use of a 3 μg dose two times for further studies on the 3–17 years population. A cohort study on healthcare workers of Brazil concluded that CoronaVac (2 doses injected nearly 3 weeks apart) is 50.7% and 51.8% effective after 2 weeks and 3 weeks, respectively, of the second dose [166]. In a Turkey-based study, serum samples of 1072 healthcare workers were collected 28 days after first dose and 21 days after second dose and assayed for SARS-CoV-2 anti-spike antibodies [167]. Antibodies of interest were detected in 77.8% of cases after the first dose and 99.6% cases after the second dose. Persons previously infected with coronavirus before vaccination displayed significantly higher (*p* < 0.001) antibody titer values than the rest. Persons with chronic disorders developed lesser antibody responses than others. The seropositive response was more frequent in females than males, and persons aged between 18–59 years presented with a higher frequency of humoral immunity. A case study in Brazil suggested that CoronaVac reduces the severity of illness after infection by P.1 variant of SARS-CoV-2, however, the argument may be raised on the sample size (2) being too small [168]. Single-dose of CoronaVac to seropositive persons with a history of COVID-19 infection produced more antibodies than a double-dose of vaccine to seronegative individuals in Indonesia [169]. In an interesting development, Calil and coworkers claimed that CoronaVac can produce specific antibodies (IgA) against SARS-CoV-2 in human milk [170]. Reduced mortality rates have been reported among healthcare workers in Turkey after the introduction of the CoronaVac vaccine [171,172]. The ratio of COVID-19 associated deaths of doctors, dentists, pharmacists, and nurses to all COVID-associated deaths reduced from 0.9% to 0.1% after vaccination.

According to phase I/II trial data of Sputnik V, all participants (*n* = 76) developed SARS-CoV-2 antibodies without any serious adverse events [90]. Interim data of 19,866 participants ((≥18 years) of the phase III trial (September–November 2020 in Moscow, Russia) were published in the Lancet [84]. The vaccine proved 91.6% effective against symptomatic COVID-19 infection with zero cases of moderate to severe COVID-19; 21 days post-vaccination. Ninety-four percent of the side effects reported were grade I, while the 4 recorded deaths were concluded to be unrelated to vaccination. In an Argentine cohort study, among 707 health professionals, a response rate of Sputnik V was 96.6%, while 71.3% reported one or more events supposedly attributed to vaccination and immunization during follow-up at 72 h post first dose [173]. In studies regarding the efficacy of Sputnik V on variants of concern, it was observed that rAd26 and rAd5 successfully neutralized S protein of B.1.1.7, showed moderate efficacy against variants with E484K mutation (also resistant to CoronaVac), and failed to neutralize S protein of B.1.351 [174,175]. Currently, the vaccine is in use in nearly 59 countries; India is one of the latest countries to approve its emergency use. In July 2021, Sputnik V was reported to maintain sera that neutralize B.1.1.7, B.1.351, P.1, and two versions of B.1.617 variants along with two more variants local to Moscow [176].

A phase I/II clinical trial regarding EpiVacCorona (NCT04527575) was conducted with 14 volunteers at stage I aged between 18 and 30 years and 86 volunteers in stage 2 aged between 18 and 60 years [92,94]. Participants were injected with two doses of EpiVacCorona 21 days apart. The injections induced specific antibody production in 100% of participants along with seroconversion with minimum neutralizing antibody titer of 1:20 after 21 days of the second dose along with zero seroconversion in the placebo group.

The developer claimed that BBV152 (Covaxin) is equally effective against mutant SARS-CoV-2 variants of concern [177]. In a study, the efficacy of BBV152 against two newly developed strains of CoV was assessed [178]. The sera from vaccinated people, as well as the sera of people who were infected with the newer strains (i.e., hCoV-19/India/20203522 and hCoV-19/India/2020Q111) were collected and cultured together to examine the neutralization in sera in vitro. The median ratio of neutralization of sera compared to mutant hCoV-19/India/20203522 strain was found to be 0.8, while it was 0.9 for hCoV-19/India/2020Q111 [178]. This experimental data demonstrates that BBV152 is effective against the infection of these two mutant strains of CoV. In a phase II clinical trial, among 921 participants screened from 7 September to 13 September 2020, 380 participants were selected with respect to the safety and immunogenicity population [179]. After 56 days, the PRNT_50_ seroconversion rates of neutralizing antibodies were found to be 92.9% (88.2–96.2%) in the 3 µg with Algel-IMDG group and 98.3% (95.1–99.6%) in 6 µg with Algel-IMDG group [179]. No significant difference or serious adverse events were reported in this study in any of the groups [179,180]. Another report from a tertiary care center in India suggested that Covaxin is safe and effective, and the adverse effects are minor in this vaccine, appearing only in 15 people out of 1322 [181].

A non-randomized, open-label phase I trial was conducted in Wuhan, China with Ad5-nCOV on 108 participants [182]. Neutralizing antibodies increased after 14 days of vaccination reaching peak levels on day 28. Antibody levels in phase I trial subjects have been claimed to remain high for six months post-vaccination according to the Chinese Centre for Disease Control and Prevention [183]. Following the trend from the phase I trial, RBD-specific antibodies increased after 14 days of vaccination, reaching peak levels on day 28 during the phase II trial [184]. Seroconversion of neutralizing antibodies to live SARS-CoV-2 occurred in 59% of cases of the middle dose group and 47% of cases of the low dose group. Relatively lower antibody response was observed in people aged ≥55 years and people with pre-existing immunity. This study also suffers from the shortcoming of a low follow-up period of 28 days.

Open-label, non-randomized phase I trial was conducted with ZyCov-D between July and October 2020 on 48 healthy volunteers ranging from 18–55 years of age [103]. Intradermal administration of three doses at day 0, day 28, and day 56 concluded that ZyCoV-D is safe, well-tolerated, and immunogenic. No deaths or serious adverse events were reported in the phase I study. Phase I/II (CTRI/2020/07/026352) and phase III trials (CTRI/2021/01/030416) have reportedly been on the way from July 2020 and January 2021, respectively; however, the results are not publicly available yet [185,186].

### 4.4. Untoward Effects

As reported by Polack et al., the occurrence of serious adverse events with BNT162b2 was low and evenly distributed between the vaccine group and placebo group: mild to moderate reactions included short-lasting pain at the injection site, fatigue, and headache [104]. There have been reports of severe allergy-like reactions after vaccination with mRNA vaccines including BNT162b2 [187,188]. Investigations suggestively point fingers at polyethylene glycols used in the formulation which have a previous history of inducing anaphylactic reactions and therefore had never been a part of any vaccine formulation before [187,189]. An Italy-based cohort study consisting of 871 volunteers reported that blood levels of anti- SARS-CoV-2 antibodies tend to decrease after 3 months of the first dose [55]. Bell’s palsy has been suspected as an adverse event related to BNT162b2. A case-control study in Israel during the first two months of 2021 reported that 56.7% of the patients hospitalized for facial nerve palsy had a recent history of vaccination with BNT162b2, meantime after vaccination being 9.3 days after the first dose and 14.0 days after the second [190] dose. The number of hospitalizations regarding nerve palsy also falls out of trend from the last 5-year data further adding fuel to the suspicion. Nevet from Israel has reported three cases of potentially life-threatening acute myocarditis within 2 days of the second dose [191]. On 9 July 2021, European medicines agency (EMA) enlisted myocarditis and pericarditis as side effects of BNT162b2 [192].

In a study on AZD1222, for Danes between 18 and 99 years old, the incidence rate of venous thromboembolism was 1.76 (95% CI 1.75–1.78) per 1000 person-years, and for Danes between 18 and 64 years old, it was 0.95 (0.94–0.96) per 1000 person-years [131]. In a study based in Norway and Denmark over the thromboembolic incidents, the results observed were the same as expected for arterial events (83 observed events against 86 expected), but more venous thromboembolic events were observed than expected in the vaccinated population (59 observed versus 30 expected) [119]. Seven of these occurrences were cerebral venous thrombosis, a life-threatening condition found to be a possible complication of ChAdOx1nCoV-19 vaccine in recent weeks [119]. Data analyzed indicated that from among the 281,264 vaccinated individuals, 7 cases are low, but still it is 20 times the general population rate and are an estimated 2.5 additional cases per 100,000 vaccinated patients [119,193]. In total, reports of adverse effects after ChAdOx1-S in the EudraVigilance database were 54,571 between 17 February 2021 and 12 March 2021. Initial adverse reactions most frequently reported were: tenderness of the injection site (63.7%), pain at the injection site (54.2%), exhaustion (53.1%), headache (52.6%), discomfort (44.0%), myalgia (44.0%), pyrexia including feverishness in 33.6% and fever >38 °C in 7.9% of the subjects [136]. Most of the adverse reactions have been mild to moderate and have been resolved within a short time after vaccination [136]. The side effects after the second dose of vaccinations were the same, but a little milder and less common [136]. A total of 28 thromboembolic reports were submitted to the database during the same period, 19 of them (67%) were by health professionals. More than half (*n* = 16; 57%) of these reports were for individuals over the age of 85; 13 (47%) were from within the EU. Of the six cases of pulmonary embolism, two were fatal, both in women, one each in the category of 18–64 years and more than 85 years of age. As well, in a male patient between 18–64 years of age as well there was a fatality following thrombosis [194]. In a case study, 18F-choline PET/CT has been evaluated for a 75-year-old male with prostate cancer and biochemical recurrence [195]. No malignancy of avid choline could be identified, but avid left axillary nodes were noted. Three days earlier, in the upper left arm, the patient received the Oxford-AstraZeneca COVID-19 vaccination followed by absorption on the left upper arm (deltoid). The nodal uptake reaction of the recent vaccination is supported by a clinical correlation [195]. The current COVID-19 pandemic vaccination scheme on 18F-choline in this case introduces a new PET/CT pitfall, which, if not noticed, may lead to the incorrect image interpretation and misrepresentation of the disease [195]. Another case study shows after 8 days of administration of COVAZD1222 vaccine, a 26-year-old woman was hospitalized for acute stroke [196]. Right hemiplegia and aphasia occurred quickly during the hospitalization of the patient for persistent nausea and headache that had begun shortly after the vaccination. Initial angiography revealed an occlusion of the left, middle cerebral artery. Dual thrombo-aspiration by using a first pass technique 3.5 h following the onset of the symptoms resulted in the rechanneling of the left middle cerebral artery following a first pass. Final angiography showed a 2C thrombolysis recanalization in the cerebral infarction. Significant thrombocytopenia, hypofibrinogenemia, and inflammation were noted in laboratory tests of this patient [196]. Roughly after a month of WHO approval, reports regarding thrombotic issues like blood clotting began to circulate after administration of ChAdOx1nCoV-19. However, following EMA, MHRA, and TGA, the WHO on 22 April 2021 in an update stated that the benefits of the vaccine outweigh the risk of blood clots [197]. In June 2021, EMA confirmed capillary leak syndrome as a potential side effect of ChAdOx1nCoV-19 [198]. In July 2021, the EMA recommended the insertion of a warning on Guillain–Barré syndrome in the product information [199].

In a phase I-IIa trial with Ad26.COV2.S involving 805 participants, local side effects like fatigue, headache, myalgia, and injection site pain were reported after vaccination, while fever was the most reported systemic adverse effect at the primary endpoint [140]. Adverse events were more common in participants receiving a two-dose schedule than those receiving single-dose, and less common in participants receiving the high dose (1 × 10^11^ viral particles/mL) than those receiving a low dose (5 × 10^10^ viral particles/mL). In Cleveland, OH, USA, a woman aged 62 years complained of altered mental status 37 days after vaccination and was ultimately diagnosed with thrombotic thrombocytopenic purpura [200]. Tests revealed elevated white blood cell count of 19.25 k/μL, absolute neutrophils 15.59 k/μL, lactate 4 mmol/L, procalcitonin 13.21 ng/mL, C-reactive protein 6.4 mg/dL, low fibrinogen 120 mg/dL, and platelets 29 k/μL. Urinalysis revealed large hemoglobin and 11–25 red blood cells per high-powered field. Nishizawa and colleagues reported a case of House–Brackmann score 4 Bell’s palsy (near-complete paralysis of the right lower face and significant paralysis of the right upper face with incomplete eye closure) after 20 days of vaccination [201]. The 62 years old Filipino woman had a history of type 2 diabetes mellitus, hypertension, and hyperlipidemia but no previous record of any neurological symptoms. Vaccine-induced thrombosis and thrombocytopenia have been reported from the USA and UK, (6 and 23 cases, respectively) between 6 to 13 days post-vaccination [200]. EMA has expressed concerns regarding the coming up of unusual blood clots as a very rare side effect of Ad26.COV2.S; however potential benefits seem to outweigh the risks [202]. Cases of vaccine-induced thrombotic thrombocytopenia have also been reported from South Africa, Canada, and many European countries [203]. Responding to such reported cases of thrombosis with thrombocytopenia syndrome, USFDA has included a new warning in April 2021 regarding rare clotting events among women aged 18–49 years; however, they are also of the opinion that potential benefits outweigh the risks [204]. A 74 years old male having known allergies to sulfa drugs and amoxicillin-clavulanic acid combinations from Virginia presented with severe cutaneous adverse reaction in the form of new-onset rash along with ipsilateral arm discomfort within 3 days of vaccination [123]. Further examination revealed erythematous plaques with acral swelling, small, non-follicular pustules spreading to the face, genitals, and mucosae without palpable lymphadenopathy. Situations improved with topical steroids and oral prednisone 20 mg. Multiple queries have been raised on the development of unusual blood clots in patients with low platelet count after vaccination with Ad26.COV2.S, however on 7 May 2021, EMA confirmed that benefits outweigh the risks [205]. Considering later developments, in July 2021 EMA enlisted capillary leak syndrome and Guillain–Barré syndrome as very rare side effects of Ad26.COV2.S in the product information sheet [206].

Adverse reactions were mostly mild to moderate and more common after the second dose in a phase I study with mRNA-1273 [145]. Trial subjects reported severe adverse events at doses ≥100 μg in another phase I study [146]. Reactogenicity symptoms like pain, redness, swelling, fever, fatigue, etc. were mild to moderate and appeared more frequently after the second dose than after the first dose in the phase III trial [74]. Local and systemic reactions after vaccination were more common in subjects aged 18–64 years than in the older subjects and disappeared within 4–5 days of vaccination in most cases. Local injection site reactions (pain, redness, swelling, axillary tenderness) and systemic adverse reactions (fever, fatigue, myalgia, headache, arthralgia, nausea, and chills) of grade 3 and above were reported by 9.1% and 16.5% of the vaccine recipients, respectively. GRADE analysis showed moderate certainty towards serious adverse events and high certainty of reactogenicity [72]. In the USA, 4,041,396 persons were injected with the first dose of the vaccine between 21 December 2020 and 10 January 2021, among which 1266 persons (0.03%) faced adverse events [207]. Among the 108 suspected cases of anaphylaxis, only 10 were confirmed as anaphylactic reactions (interestingly, all were females aged between 31–63 years), 5 of which had a previous history of anaphylaxis, and 9 had a history of allergic reactions. While symptoms of anaphylactic shock appeared within 45 min, no anaphylactic death was recorded. Forty-seven of the remaining suspected cases were concluded to be non-allergic reactions while forty-seven were allergic reactions not related to anaphylaxis and four cases were abandoned due to lack of sufficient information. The occurrence rate of anaphylaxis was found to be 2.5 per million vaccinations which is higher than the rate (1.3 per million vaccinations) with other similar vaccines [208]. In the phase III CoV efficacy trial of this vaccine, out of 15,185 recipients of the first dose, 228 (1.5%) reported hypersensitivity-associated adverse events [209]. Among them, 4 cases had very specific symptoms of localized erythematous rash surrounding the injection site within a few days of the first dose, often referred to as ‘COVID arm’. However, the ‘COVID arm’ has been perceived as a harmless side effect by physicians [172]. All 4 reported cases were in females, which is in conjunction with findings by [204] fuelling the possibility of further clinical analysis regarding any relationship between sex and occurrence of hypersensitivity reactions after administration of the mRNA-1273 vaccine. A 22 years old myopic female in the USA experienced bilateral retinal detachment after vaccination with mRNA-1273 [210]. In July 2021, EMA confirmed myocarditis and pericarditis as side effects of mRNA-1273 [211].

Xia and colleagues reported only mild pain at the injection site and fever as adverse events after phase I and phase II trials with BBIBP-Cor-V in China [153]. Adverse events were mostly mild to moderate among recipients of BBIBP-CorV, the most common reaction being fever as reported from phase I and phase II clinical trials conducted by the Beijing Institute of Biological Products (Beijing, China) [212]. Jordanian healthcare workers receiving BBIBP-CorV reported mild side effects like injection site pain, fatigue, fever, myalgia, headache, dizziness, arm numbness, and ear symptoms within 0–5 days [213]. The occurrence of side effects was more after the second dose, while no statistically significant relationship could be established between severity of side effects and sex or age group.

In a phase I/II trial of CoronaVac, while most of the adverse effects were categorized as mild to moderate with pain at the injection site being most frequent, 2% of the recipients reported serious adverse events which were judged to be unrelated to vaccination [145]. One serious adverse event recorded was pneumonia in the placebo (alum only) group in a China-based trial on the juvenile population [149]. An independent cross-sectional study was undertaken for CoronaVac side effects among Turkish healthcare workers in February 2021 [214]. Pain at the site of injection was the most frequent side effect (41.5%) within 4 weeks of vaccination while fatigue, headache, muscle pain, and joint pain followed later. Females were more prone to side effects than males. The study concluded CoronaVac to be safe overall, however, younger age, previous infection history, and compromised health status seemed to enhance the risk of side effects. A case report stated the development of SDRIFE like eruptions and itchy rashes 4 days after CoronaVac in an 87-year-old patient, though the specific allergen could not be identified yet [215]. In a 45-year-old female healthcare worker of Turkey, pityriasis rosea (skin rashes, herald patch, multiple plaques in chest region), generally caused by the herpes virus and rarely caused after vaccination developed 4 days after both the first and the second dose of CoronaVac which cured within few weeks on treatment [216]. Though when compared to life threats skin eruptions are somewhat acceptable, close monitoring may be recommended as the mechanism behind the development of this side effect is not clear yet. A Thailand-based clinical trial (ictrp-TCTR20210610004) has reported neurological complications after CoronaVac injection [217]. Type I Kounis syndrome, pustular psoriasis, petechial skin rash, and reactive arthritis are among other adverse effects reported after CoronaVac administration [218,219,220,221].

In May 2021, a Brazilian health regulatory agency alleged contamination of Sputnik V, claiming the second dose contains adenovirus capable of replication and a posing potential risk for the recipients [222]. An Argentine cohort study supported short-term safety from early serious events but displayed high rates of local and systemic reactions [173]. Muscle pain, injection site pain, fever, redness, swelling, and diarrhea were the highest reported local and systemic adverse events. Occurrences of adverse events were higher in females and in the age groups below 55 years. A recent infodemiology study by Jarynowski et al. [223] claims Sputnik V to cause mild to moderate adverse effects in females compared to males and increase in number with increasing age (0.05/year, *p* < 0.001). Reported adverse events were in line with other vector vaccines including fever, pain, fatigue, and headache. A cohort study by the Republic of San Marino analyzed data of 2558 persons (between 18–89 years); 7 days after the first dose and 1288 people after 7 days of the second dose [224]. After the first dose, vaccine recipients described both local and systemic reactions in 16.4% cases, 25.8% reported systemic reactions only and 10.2% reported local symptoms only whereas after the second dose, both local and systemic reactions were reported in 31.9% cases, 18.5% reported systemic reactions only and 16.1% reported local symptoms: only chief complaints being injection site pain, asthenia, headache, and joint pain. 81.8% of the people experienced adverse events after both the first and the second dose. Ryzhikov and colleagues reported mild pain at the injection site as the only adverse effect of EpiVacCorona [92].

In a randomized control trial of BBV152 with 375 participants, three groups were formed with 100 participants in each of them randomly selected for all three vaccine groups, and 75 were allocated randomly for the control group receiving Algel only [178]. After injection of both the doses, 17 (17%; 95% CI 10.5–26.1%) participants in the 3 μg group with Algel-IMDG, 21 (21%; 95% CI 13.8–30.5) in a 6 μg group with Algel-IMDG, and 14 (14%; 95% CI 8.1–22.7) in the 6 μg group with Algel group reported local or systemic adverse reactions [178]. Injection site pain (5% of total participants), headache (3%), fatigue (3%), fever (2%), and nausea/vomiting (2%) were the most reported adverse events out of which 69% were mild and 31% were moderate [178]. Srivastava and colleagues claimed that at a particular tertiary care center in India, only 15 candidates out of 1322 were found to have minor adverse effects after the first dose of BBV152 between 16th January 2021 and 16th February 2021 [181]. The adverse effects included pain at the injection site, chest pain, fever, headache, etc.

As reported by Zhu and colleagues regarding Ad5-nCoV, 83% of the low dose group (1 vial of 5 × 10^10^ viral particles per 0.5 mL), 83% of the middle dose group (2 vials of 5 × 10^10^ viral particles per 0.5 mL) and 75% of the high dose group (3 vials of 5 × 10^10^ viral particles per 0.5 mL) reported at least one adverse reaction within 7 days of vaccination [182]. Most common local and systemic adverse effects included pain at the injection site, fever, fatigue, headache, and muscle pain, all with mild to moderate severity, while no serious adverse events were recorded within 28 days of vaccination. However, the narrow age range of the volunteers, short follow-up period, and absence of any randomized control pose doubts on the acceptability of the outcomes. A placebo-controlled, randomized, double-blind phase II clinical trial was also conducted by Zhu and colleagues on 508 adult volunteers divided into three groups viz. low dose (1 × 10^11^ viral particles per mL), middle-dose (5 × 10^10^ VP per mL) and placebo [184]. 14 days post-vaccination, significantly higher reports of adverse events were reported from low and middle-dose populations (74% and 72%, respectively) than placebo recipients. Adverse events were similar to those reported in the phase I trial and the joint pain was the lone additional symptom [182,184]. Nine percent of the middle dose group reported severe adverse events.

## 5. Vaccines under Development: A Brief Overview of Current Forerunners

The global pandemic has already affected innumerable lives in many ways. Vaccines are the need of the hour to prevent the loss of human resources and the economy. Coalitions for economic preparedness innovations, global alliances for vaccines and immunization, and WHO are collaboratively leading COVAX in partnership with UNICEF. COVAX is a global sharing platform aiming to accelerate the development and manufacture of COVID-19 vaccines worldwide and subsequently ensure equitable distribution of vaccines worldwide. Operation warp speed, a public-private venture led by the US government, was announced in May 2020 to speed up the development, manufacturing, and distribution of COVID-19 vaccines. These two initiatives have played havoc with the release of many of the vaccine candidates. Table 3 enlists the current frontrunners in clinical trials likely to get approval in the near future.

A US phase I trial consisting of persons with age groups of 18–55 years and 65–85 years recommended BNT162b2 over BNT162b1 for further trials based mainly on lesser systemic reactogenicity among the older population [225,226]. Vaccine BNT162b1 is still in the final phases of the clinical trial while BNT162b2 is already approved by WHO. China developed a recombinant vaccine candidate using Sf9 cells, which exhibited promise in clinical trials (currently in phase III, NCT04904471) [227]. A lipid nanoparticle-encapsulated mRNA vaccine candidate coding for the RBD developed by Walvax Biotechnology Co. Ltd., Kunming, China (ARCoV) is all set to start a phase III clinical trial (NCT04847102) [228]. A prefusion stabilized protein nanoparticle vaccine candidate, NVX-CoV2373, has already been granted provisional determination by TGA and fast track designation by USFDA. Reportedly, NVX-CoV23, demonstrated 51.0% efficacy against the B.1.351 variant of CoV in HIV-negative subjects [229]. Medicago has developed a plant-based adjuvant vaccine candidate (CoVLP) belonging to virus-like particles currently undergoing phase III clinical trial (NCT04636697) [230]. The vaccine candidate has displayed high antibody titers compared to natural infection according to phase II results made public on 18 May 2021 through a press release by Medicago and GSK [231]. USFDA has granted fast track designation for the vaccine candidate in February 2021 [232]. Valneva has developed an inactivated vaccine candidate VLA2001 claimed to be safe and well-tolerated to human subjects in a press release dated 6 April 2021 [233]. A phase III study (NCT04864561) comprising about 4000 participants aiming to compare VLA2001 with AZD1222 is on the way starting from April 2021 [234]. Biological E, India in collaboration with Dynavax (Emeryville, CA, USA) and Baylor College of Medicine (Houston, TX, USA) has developed a protein subunit-adjuvant complex vaccine candidate specifically aimed at children. The candidate is all set to enter into the 3rd phase of the clinical trial after very positive results in earlier phases (CTRI/2020/11/029032) in India [235]. Sanofi and GSK have announced 95–100% seroconversion in interim phase II results on 17 May 2021 with their vaccine candidate Vidprevtyn comprising recombinant protein and adjuvant [236]. Phase III trial (NCT04904471) was begun in May 2021 and EMA started a rolling review for the candidate in July 2021 [227]. Vidprevtyn is expected to be effective against D.614 and B.1.351 variants of CoV according to a press release by Sanofi on 27 May 2021 [237]. Nanogen Pharmaceuticals, Vietnam has developed a glycosylated recombinant S protein vaccine candidate, nanocovax, against SARS-CoV-2. The vaccine candidate demonstrated significant neutralizing activity a week after two doses. In a phase I/II clinical trial, nanocovax exhibited a good safety profile and most of the adverse effects were grade 1, which disappeared within 48 h of injection. [238]. Phase III study (NCT04922788) was begun in June 2021 with the candidate [239]. A CoV fusion protein vaccine candidate (V-01) has been developed in China which is able to trigger an immune response in addition to demonstrating a good safety profile in phases I (ChiCTR2100045108) and II (ChiCTR2100045107) [240,241]. A recombinant S protein vaccine candidate, Razi CoV Pars is showing promising results. Phase III clinical trial (IRCT20201214049709N3) has already started with about 41,000 participants in Iran [242]. GBP510, a nanoparticle vaccine candidate displaying key regions of S protein being used in combination with an adjuvant, is coming up with promising results in the Republic of Korea. The one with adjuvant ASO3 has been proven as safe and immunogenic during the first two phases of the human trial (NCT04750343) [243]. Phase III trial comparing GBP510 with AZD1222 has been approved to start in the Republic of Korea (NCT05007951) [244]. Attempts are also being made towards passively acquired immunity by the use of plasma therapy, monoclonal antibodies, and cocktail antibodies [60,245]. According to TASS News Agency, Russian Academy of Science has developed an inactivated virus vaccine candidate, CoviVac which is claimed to be suited as a secondary immunogen/booster effective against most mutants of novel CoV [246].

## 6. Possibility of Reinfection: What Goes Around, Can Come Back Around?

All vaccines approved by regulators for public use have proven themselves to protect the vaccinated subject from COVID-19 in clinical trial setups. Vaccination is expected to prevent severe illness, hospitalization, and death resulting from SARS-CoV-2 infection. However, from a bird’s eye viewpoint, no one is completely protected until everyone is protected. Vaccination is a very important tool to deal with the current pandemic. While the vaccines in use are effective, no vaccine is able to prevent infection 100% of the time. However, there is a fine delineation among reinfection, relapse, and positivity which should not be misinterpreted. The emergence of new mutant strains of CoV makes the situation more complicated. How frequently vaccine breakthrough cases are coming up is an indication of the safety of the vaccinated population from SARS-CoV-2. Edridge and peers [247] reported reinfection by seasonal human CoVs as early as within 6 to 12 months of the first infection.

A person could be infected just before or just after vaccination and get sick. It typically takes a few weeks for the human immune system to build up protection after vaccination, hence a person may get sick if the vaccine has not had enough time to provide protection. However, the severity of the disease would be low in a vaccinated individual. In most cases of reinfection, the first episode of the disease was mild or asymptomatic [248]. The greater magnitude of antibody responses and T-cell responses generated during severe COVID-19 may confer more robust and long-lasting protection.

Researchers are trying to extract a pattern or trend from the breakthrough cases based on age, sex, health condition, race, vaccine type, viral strain, etc. Vaccines can either block new infections or can halt the progression of symptoms after infection by stimulating the immune system. Protection seems to be dependent upon antibody response along with important contributions from CD4+ T cells [249]. Neutralizing antibodies generated after infection or vaccination last for months to years but are not lifelong [250,251]. There still exist remote chances of infection of CoV back from humans to animals, which may potentially induce vaccine failure [252]. Neutralizing antibody titer seems to decrease 8 folds within 144 days of natural infection in a patient reinfected with COVID-19 [13]. During the second infection, antibody response increased continuously from day 3 to day 8 of hospitalization while IgM response was absent.

## 7. Stories of Some Unsuccessful Vaccine Development

Under the unforeseen circumstances of the COVID-19 pandemic, numerous organizations attempted to develop effective vaccines around the world. Though some of them have already made the cut and some more are likely to do so in near future, many attempts have failed to travel the path of success. Institut Pasteur and the University of Pittsburgh (Pittsburgh, PA, USA) developed a viral vector vaccine against COVID-19, V-591 (TMV-083) using Measles virus Schwarz vaccine strain as vector expressing S protein of SARS-CoV-2 [253]. IAVI Merck (Merck Sharp & Dohme Corp., Kenilworth, NJ, USA) developed another viral vector vaccine, V-590, using replication-competent vesicular stomatitis virus expressing S protein of SARS-CoV-2. In phase I clinical trials, both the vaccine candidates exhibited good tolerability [254,255]. However, owing to their inferior immune response compared to other vaccine candidates under development, Merck announced plans to discontinue their production [256,257]. Imperial College London (London, UK) developed a self-amplifying RNA vaccine aiming to target viral mutations. Though the developers expressed cautious optimism, they decided to abandon the Phase III trials later and focus on developing improved vaccines [258]. The University of Alabama (Tuscaloosa, AL, USA), Birmingham in collaboration with Altimmune Inc. (Gaithersburg, MD, USA) and Summit Biosciences (Lexington, KY, USA) developed an intranasal COVID-19 vaccine, AdCOVID, which has been designed to express RBD of the SARS-CoV-2 S protein. AdCOVID exhibited exciting preclinical results in neutralizing serum activity and boosting potent immunity (IgA) in the respiratory mucosa in mice [259]. However, due to inadequate immune response as observed in phase I clinical trial (NCT04679909), further development of AdCOVID was suspended [260]. MRT5500, a double mutant RNA vaccine candidate expressing S protein (2P/GSAS S mRNA) of SARS-CoV-2 developed under collaboration between Sanofi Pasteur and Translate Bio, which exhibited potential immunogenic response by activating neutralizing antibodies and Th1-mediated immune response in preclinical studies [261]. Owing to preclinical success, the developer initiated Phase I/II clinical trial (NCT04798027) of MRT5500 [262]. In phase I/II clinical trial, MRT5500 displayed neutralizing antibody seroconversion (defined as 4-fold rise vs baseline) in > 90% participants two weeks after the second dose (2 doses 21 days apart) regardless of any safety and tolerability issue. However, Sanofi Pasteur announced to discontinue further trial with mRNA COVID-19 vaccines and to focus more on the recombinant vaccine, developed in collaboration with GSK [263]. Recently, another mRNA vaccine, H0005845 (CVnCoV) has been withdrawn by CureVac AG. In a letter to the European Medicines Agency, the developer stated that they would like to focus more on COVID-19 vaccine development as part of a second generation vaccine program. However, they would like to continue the ongoing clinical trials [264]. In March 2021, eight European countries have so far entirely suspended the use of Oxford-AstraZeneca AZD1222 vaccine over concerns regarding possible side effects including blood clots. However, according to WHO and EMA, there is no such association between the AZD1222 vaccine and complaints of blood clots [197,265]. The University of Queensland (Santa Lucia, QLD, Australia) and CSL developed a protein subunit vaccine candidate, v451 which exhibited a significant response to the virus and displayed a good safety profile in phase I clinical trial [266]. Phase I data showed that the production of antibodies is directed against fragments of gp41 protein, which is an ingredient used to stabilize the vaccine [267]. The risk of a partial immune response to this gp41 was intimated to the trial participants and the extent of this immune response would not interfere with HIV tests [267]. The University of Queensland further stated that there is no chance of getting HIV infection, and routine follow-up tests also confirmed that the HIV virus is absent in vaccine recipients [267]. However, the University of Queensland, CSL, and the Australian Government have agreed to halt this vaccine research for phase II/III trials, citing the need for significant modifications in well-established HIV testing protocols in the healthcare sector before moving further with the project [267]. In April 2021, the University of Queensland undertook a new re-engineering project to circumvent the shortcomings of previous development [268]. The Iranian government has recently decided to abandon the production of their home-grown (Organization of Defensive Innovation and Research, Iran) COVID-19 vaccine, Fakhravac (an inactivated viral vaccine), citing reduced demands compared to imported vaccines [269]. The failed attempts may be seen as learning trials to obtain the correct gateway. The pauses and setbacks are not very rare, instead of demotivation; they would rather generate assurances that the human health issue will not be compromised.

## 8. Discussions: Lessons Learned towards Future Directions

Many vaccines have been developed against COVID-19 so far using different platforms. Probably for the first time in history, companies have started scaling up the production capacity even before getting approval foreseeing the huge demand post-approval. The requirement of a cold chain is proving to be a key factor towards mass vaccination in a multi-centric mode. Exact antibody titers or defined T cell responses are yet to be arrived at to guarantee protection from SARS-CoV-2. There are no set international standards regarding these parameters also, which might result in different interpretations and approval in different countries from similar outcomes. The durability of the induced immunity is still under research [270]. Until now, very few data are available on the effect of vaccines on vulnerable populations like pregnant ladies, children, infants through lactating women, pre-existing disease conditions, immunodeficiency, etc. The success of vaccination largely depends on virus variants and host immunity. It is also to be made clear that the ‘best’ COVID-19 vaccine is nothing but a utopian maxim since different vaccines use different technologies and different platforms, each of which might suit the appropriate population under proper contexts.

Though the fast-tracking of the vaccine candidates seems justified under the current pandemic emergency, it is to be taken care that no aspect of potential risks gets overlooked in undue haste. The viral genome is prone to mutations by antigenic shift and antigenic drift while spreading through different populations in different environmental conditions [271]. This may in turn give rise to a resistant strain with time. Too much stress on the S protein as the target antigen may generate a mutation force. The vaccine might then become seasonal protection like in the case of influenza. Apart from vaccine development, the success of strategic herd immunity through vaccination is largely dependent on the proper distribution of the vaccines throughout the globe also.

**Figure 3:**
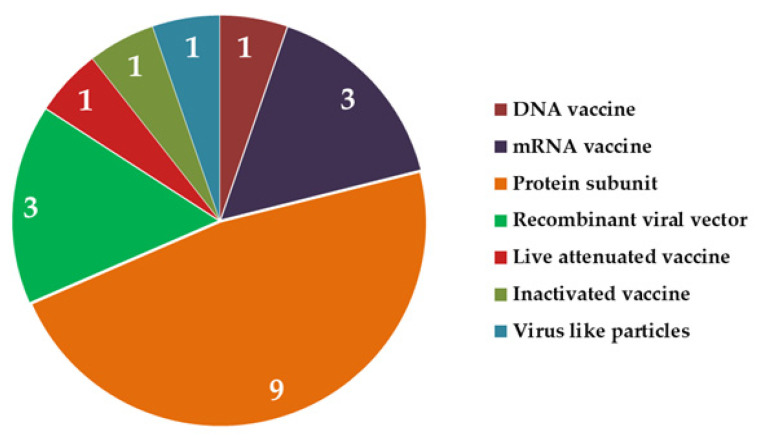
Vaccines currently in the final phase of clinical trial. Protein subunit vaccines (9) dominate the list followed by mRNA vaccine and recombinant viral vector (3 each). Each of live attenuated, inactivated, virus like particles, and DNA vaccines are in the final phase trial.

**Figure 4:**
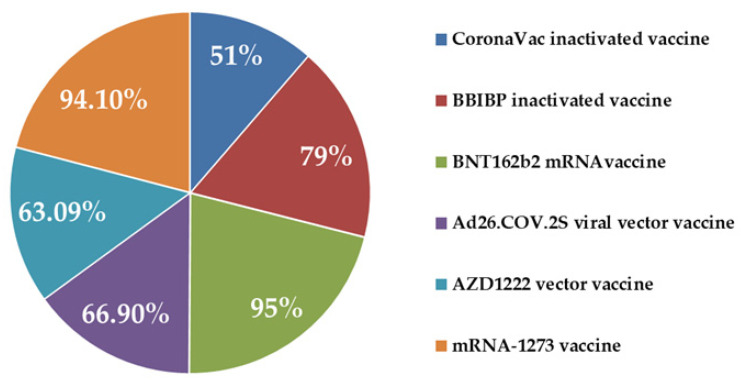
Percentage efficacy of WHO approved vaccines against symptomatic COVID-19.

Results and data regarding COVID-19 vaccines are mostly available from the manufacturers and from governments of various countries, rather than from peer-reviewed publications now. Both BNT162 and mRNA-1273 possess the unique advantage of possible quick re-engineering if needed to cope up with new mutations of CoV [272]. There exist multiple pieces of evidence that mRNA COVID-19 vaccines (Pfizer-BioNTech, Moderna) offer similar protection in real-world conditions [273,274,275]. Some vaccines, particularly the inactivated vaccines and subunit vaccines, suffer from very short immune memory [49]. From the safety point of view, clinicians are actively considering the possibility of recommending mRNA vaccines instead of viral vector vaccines for those at substantially higher risks of TTS [276]. Interestingly, capillary leak syndrome and Guillain–Barré syndrome have been reported as very rare side effects of both AstraZeneca and Janssen as listed by EMA. Interestingly, Guillain–Barré syndrome is also enlisted as a post-infection complication related to CNS [277,278]. In a comparative survey on Pfizer-BioNTech and Moderna vaccines (both mRNA vaccines), both of them presented with almost identical efficacy profiles regarding protection against COVID-19, while the former one came up with a lesser number of reported adverse effects whereas the latter one seemed more convenient regarding transport and storage conditions [279]. Statistical analysis of results concluded that Sinopharm, Oxford-AstraZeneca, and Pfizer-BioNTech vaccines provided a similar level of protection from COVID-19 [158]. Wang and colleagues have described both BBIBP-CorV and CoronaVac to be more or less equally effective against the B.1.1.7 (alpha) variant, but they might be less effective against the B.1.351 (beta) variant local to South Africa [280]. Variants with mutations in the RBD were effectively neutralized by CoronaVac (inactivated virus vaccine) but variants with E484K mutation were resistant to CoronaVac [162]. Comparatively low efficacy of the two-dose schedule of CoronaVac than single dose was doubted to be the result of some non-pharmaceutical interventions or bias in the setting [163]. Two doses of CoronaVac have been observed to boost longer-lasting neutralizing antibody responses in previously seropositive patients possibly via inducing B cell memory responses [281]. However, this boosting effect was absent in three patients, obesity being the common factor among them. In the same study, it was also noted that both CoronaVac (2 doses) and BNT162b2 (single dose) yielded similar neutralizing antibody responses to naturally infected patients between 4.2 and 13.3 months of vaccination. In Indonesia, COVID-19 symptoms developed 40 days post-vaccination with CoronaVac in a patient (female, 41 years) on 19 March 2021 [282]. Though the patient likely had a limited amount of humoral immune response to the vaccine, no history of immunodeficiency disorders has been found. Moreover, antibody level in this patient was much lower than those previously infected with COVID-19. Interestingly, a high titer of the antibody was present 20 days after the development of symptoms. Observations hint towards either lack of immune response elicited by the vaccine and subsequent infection with the variant virus or involvement of a different variant of the virus resistant to CoronaVac. It has been observed that rAd26 and rAd5 successfully neutralized S protein of B.1.1.7, showed moderate efficacy against variants with E484K mutation (also resistant to CoronaVac), and failed to neutralize S protein of B.1.351 [174,175]. A network meta-analysis provided the order of effectiveness as BNT162b2 ≃ mRNA-1273 > Sputnik V >> AZD1222 [283]. Concerning the untoward effects arising from vaccination, the risk-benefit balance must favor the latter. Table 4 enlists the reported adverse reactions of the vaccines currently in use. The majority of the approved vaccines are represented by viral vector vaccines while among the frontrunners in the pipeline, protein subunit vaccines gained an overwhelming majority (Figure 3). The mRNA vaccines have displayed very high efficacy against symptomatic COVID-19 infection, while inactivated vaccines and viral vector vaccines perform better to prevent COVID-associated hospitalization (Figure 4).

The temporary lack of peer review is somewhat posing doubts on some questionable results being presented among the scientific fraternity. Reuters expressed doubt on the claimed immunogenic response by the vaccines as many of the vaccine candidates failed to exhibit a similar level of antibody generation in trials than claimed by their developers [284]. The scientific community worldwide is still divided on whether or not the peptides used in EpiVacCorona are detectable to the human immune system to generate B cell response [285,286,287]. A report published in the Lancet claims that the vaccine BBV152 has been rolling out even though there are several trial allegations made against them [288]. The allegations included participants claiming that they were unable to read the consent form and they were unable to report adverse events. Participants also claimed that they did not know it was a trial and they could refuse to take the vaccine [288].

In a study to compare two vaccines AZD1222 and BBV152, the first dose of vaccines was injected into 552 healthcare workers, where 456 received AZD1222 and 96 received BBV152 [289]. In total, seropositivity after the first dose was found to be 79.3%. In AZD1222, responder rate and median (IQR) rise in anti-S protein, antibody was significantly higher compared to BBV152 (86.8% vs. 43.8%; 61.5 AU/mL vs. 6 AU/mL; both *p* < 0.001) [289]. No differences were observed with respect to age, gender, and BMI. There was a lesser response rate in the subjects with a history of hypertension (65.7% vs. 82.3%, *p* = 0.001). The recipient of AZD1222 had more negative effects compared to recipients of BBV152 (46.7% vs. 32.4%, *p* = 0.006). In a randomized controlled study, of the 515 healthcare workers, 95.0% showed seropositivity following both doses of the AZD1222 and BBV152 [273]. The 98.1% and 80.0%, of the participants, showed seropositivity among the 425 AZD1222 and 90 BBV152 recipients, respectively. In line with the observations after the first dose, the seropositivity rate and median rise in anti-spike antibodies were higher in AD1222 compared to those in BBV152 (98.1% vs. 80.0%; 127.0 AU/mL vs. 53 AU/mL; both cases *p* < 0.001). Though there was no definitive agreement observed with regard to sex, BMI, and blood group, there were significantly lower seropositive rates for either people >60 years of age or people with type 2 diabetes [290]. Recipients irrespective of the vaccine type received had mild to moderate adverse effects which were similar and expected, and neither had any serious unwanted effects [290]. In June 2020, China’s Central Military Commission gave permission for use of Ad5-nCoV by the military for 1 year, which is arguably equivalent to a Phase III trial [73,291]. Both Oxford/Astrazeneca and CanSino utilize adenovirus as a vector for their COVID-19 vaccine. Adenoviruses are common and can cause a variety of illnesses in humans ranging from a cold to conjunctivitis. When comparing the neutralizing antibody response between the two-adenoviral vector-based vaccine candidates, it was shown that while Oxford/AstraZeneca’s AZD1222 has demonstrated a high neutralizing antibody level in 91% of individuals following the first dose, and in all individuals following a booster dose, only 59% of individuals in CanSino’s vaccine demonstrated neutralizing antibodies [134,181]. This indicates that a good proportion of participants did not develop an effective immune response due to the presence of pre-existing immunity against human adenoviruses. Oxford/AstraZeneca was able to prevent this outcome by utilizing a genetically modified chimpanzee-derived adenovirus against which humans do not have pre-existing immunity [73]. However, CanSino’s vaccine, at a lower cost combined with its moderate efficacy, may prove advantageous for some countries.

Despite that the vaccination has been regarded as the first-line measure to prevent and slow down SARS-CoV-2 infection, scientists are still looking forward to developing other therapeutic strategies to treat COVID-19 disease. Going against the trend of new vaccine developments, Merck has come up with an anti-COVID pill, a molnupiravir capsule with potential of becoming the first anti-COVID-19 oral formulation aimed at keeping people out of the hospital [292]. Molnupiravir resembles ribonucleosides and gets incorporated into viral RNA producing defective constructs to impair viral replication [292]. The medicine seems to have a low risk of serious side effects [292]. Moreover, it is effective against mutant variants of concern [292]. Despite a temporary halt in clinical trials, the molnupiravir pill has been proven to be effective in reducing the risk of hospitalization or death to 50% of patients with mild to moderate COVID-19 disease compared to placebo in phase III study [293]. Merck is optimistic about obtaining USFDA approval for emergency use authorization of the pill [292].

## 9. Conclusions

Ideally, the vaccine needs to be made available to nearly 8 billion people worldwide. Safe and equitable distribution is a challenge, especially in developing and under-developed countries. It takes around 2–4 weeks post-vaccination to develop immunity. Initially, the healthcare workers, susceptible aged population with co-morbidities, and potential super spreaders had been prioritized for vaccination. Currently, the entire world is racing to get to the end of the pandemic. The durability of the elicited immune response is a critical player to generate sustainable herd immunity worldwide. To further mobilize people towards vaccination, the principle of a vaccine passport is being actively considered by some countries. Different technologies, platforms, and routes have been utilized to develop safe and effective vaccines. Choice of optimum vaccine for a population sub-group depends on factors like race, environmental conditions, prevalent immunity, health conditions, etc. Ideal interval and dose for vaccination to naturally infected persons post-infection are also matters of concern. Moreover, mutation of the highly contagious retrovirus is a headache. All approved vaccines are more than 50% efficacious in preventing COVID-19. Some are displaying promise against newer strains also. The delta strain (B.1.617.2) has been in discussion for quite a bit now regarding the future third wave. Sputnik V has displayed high efficacy against B.1.617.2. The BBIBP-CorV has also demonstrated a similar antibody response against B.1.617.2 compared to natural infection. Both Ad26.COV2.S and BNT162b2 have also demonstrated some efficacy against B.1.617.2. Many candidates are showing promise in clinical trials, and are likely to be approved in the future. As soon as the world population becomes vaccinated, the pandemic will start to fade out, as no one is safe until everyone is safe.

## Figures and Tables

**Table 1:** Summary of SARS-CoV-2 vaccine strategies with their advantages and limitations.

S. No.	Strategies	Advantages	Limitations	Leading Candidates
1	DNA vaccine	Stable, cost effective, induces cellular, humoral and neutralizing antibody response	Immunogenicity lower than viral vaccines	Zycov-D, INO-4800
2	mRNA vaccine	Easy to design, lower risk of accidental infection than viral vaccines	Carrier required to stabilize and pack naked RNA	BNT162b2, mRNA-1273, ARCoV, CureVac (Tübingen, Germany)
3	Protein subunit vaccine	Non-infectious, pure antigens easily elicit immunogenic response	Comparatively costlier	Corbevax, Sanofi (Paris, France) NVX-CoV2373, UB612, SCB-2019, EpiVacCorona (Federal Budgetary Research Institution State Research Center of Virology and Biotechnology, Koltsovo, Russia), Nanocovax (Nanogen Pharmaceutical, Ho Chi Minh City, Vietnam)
4	Recombinant viral vector vaccine	Efficient design easily elicits immunogenicity to desired level, fast and reusable platform	Possibility of undesirable reactions, possibility of Th2 bias	AZD1222, Janssen (Bersee, Belgium), Immunity Bio (Culver City, CA, USA), GRAd-COV2, Sputnik V (Gamaleya Research Institute, Moscow, Russia), Convidicea (Cansino Biologics, Tianjin, China), OraPro-COVID-19™ (IosBio, Somarset, UK and Biocell Corporation, Auckland, New Zealand)
5	Live attenuated vaccine	Presents entire viral antigen to immune system, strong and long-lasting immune response	Risk of infection going out of control, not suitable for immunocompromised individuals	Covi-Vac, BCG (repurposing)
6	Whole killed vaccine	Rapid development, can elicit very good immunogenic response, broad antigenic profile	Th2 bias	BBIBP-CorV, CoronaVac, VLA2001, BBV152
7	Virus like particles vaccine	Non-infectious, broad antigenic profile	Weaker immunogenicity	Medicago (Quebec City, Canada)

**Table 2:** A summary table of the vaccines approved by WHO.

S. No.	Vaccines	Types	Carriers	Doses
1	Oxford-AstraZeneca (ChAdOx1nCoV-19, AZD1222) (University of Oxford, Oxford, UK)	Viral vector, targeted towards S protein	Modified Chimpanzee Adenovirus ChAdOx1	2 doses 8 to 12 weeks apart, i.m.
2	Pfizer-BioNTech (BNT162b2)	Nucleoside modified mRNA	Lipid nanoparticles	2 doses 21 to 28 days apart, i.m.
3	Johnson and Johnson (Ad26.COV2.S, Janssen)	S protein of SARS-CoV-2 WA1/2020 strain	Recombinant, replication incompetent adenovirus Ad26	Single dose, i.m.
4	Moderna (mRNA-1273)	Nucleoside modified mRNA	Lipid nanoparticles	2 doses, 4 to 6 weeks apart, i.m.
5	Sinopharm (BBIBP-CorV)	Inactivated virus (2019-CoV)	Inactivated virus + adjuvant	2 doses, 3–4 weeks apart, i.m.
6	CoronaVac (Sinovac)	Inactivated virus	Inactivated virus + adjuvant	2 doses, 2–4 weeks apart, i.m.

i.m., intramuscular.

**Table 3:** Forerunners in clinical trials: possible candidates for future.

S. No.	Vaccines	Country of Origin	Trial Phase	Types	Potential Promises
1	Vidprevtyn	USA	III	Recombinant protein with adjuvant	Interim phase II result reported 95–100% seroconversion, 3rd phase trial started in May 2021
2	CVnCoV (CureVac)	Germany and Belgium	IIb/III	Non-chemically modified mRNA (CBnCOV)	Protection against B.1.951 variant in mice, 48% efficacy in phase IIb/III trial
3	BCG vaccine (repurposing)	Australia, The Netherlands	II/III	Live attenuated virus	Reduced COVID-19 related clinical symptoms, not impressive enough for confirmatory decision
4	NVX-CoV2373	Australia	III	Protein nanoparticles	About 90% efficacy reported in various trials, approval sought in Australia, USA, Canada, Europe
5	ARCoV	China	III	mRNA (encoding receptor binding domain) lipid nanoparticles	Phase III trial on the way
6	Unnamed (Medicago)	Canada	III	Virus like particle along with plant based adjuvants	High antibody titers with tolerable safety profile
7	VLA2001	UK	III	Inactivated vaccine	Safe, well tolerated as per phase I/II trial reports in April 2021
8	Corbevax	India	III	Protein subunit with CpG1018 as adjuvant	Very positive results from phase I/II, 3rd phase announced on April 2021
9	Nanocovax	Vietnam	III	Glycosylated recombinant S protein	Displayed in vitro neutralizing activity, in phase II, all non-placebo recipients developed antibodies
10	BNT162	USA	I/II/III	mRNA vaccine	BNT162b2 already approved by WHO, BNT162b1 displayed similar efficacy with altered adverse reaction profile
11	INO-4800	USA	II/III	Intradermal DNA vaccine (plasmid)	Phase II portion declared INO-4800 as safe and well tolerated in May 2021
12	Unnamed (Immunity Bio, Culver City, CA, USA)	USA	II/III	Adenovirus based vaccine targeting S protein and nucleocapsid DNA	Reported CD4+ and CD8+ antigen specific T cell response in mice, no serious adverse events reported in human receiving low dose
13	UB612	Taiwan	II/III	Multitope peptide vaccine	Well tolerated, CD4+/CD8+ T cell response
14	GRAd-COV2	Italy	II/III	Defective gorilla adenovirus encoding prefusion stabilized full length S protein of SARS-CoV-2	Reported as safe and well tolerated in phase I on November 2020
15	SCB-2019	China	II/III	Protein subunit with adjuvants	Adjuvant optimized for formulation, robust cellular and humoral immune response reported along with strong neutralizing activity
16	Unnamed (West Bank Biopharma)	China	III	Recombinant vaccine (Sf9 cells) targeting receptor binding domain using Baculovirus vector	3rd Phase trial is enrolling by invitation
17	V-01	China	III	Recombinant fusion protein	Induces immune response, good safety profile in phase 2 trial
18	Razi Cov Pars	Iran	III	Recombinant S protein	Phase III trial started after potential promise shown in earlier phases
19	GBP510	Korea	III	Nanoparticles (key regions of viral S protein)	Phase III trial approved based on immune response and safety profile in earlier phases

**Table 4:** Reported adverse effects of approved vaccines with respect to vaccine types.

S. No.	Vaccine Types	Approved Vaccines	Adverse Events	
			Mild	Severe
1	mRNA vaccines	BNT162b2, mRNA-1273	Pain, tenderness, redness and swelling at injection site, fatigue, headache, fever, nausea, chills, COVID arm	Allergy-like reactions, Bell’s palsy, acute myocarditis, pericarditis, arthralgia (grade 3 and above), anaphylactic reactions, bilateral retinal detachment
2	Protein subunit vaccines	EpiVacCorona	Pain at injection site	-
3	Recombinant viral vector vaccines	AZD1222, Ad26.COV2.S, Sputnik V, Convidicea	Pain and tenderness at injection site, exhaustion, discomfort, headache, pyrexia, fatigue, muscle pain, diarrhoea, asthenia, joint pain	Clotting events, venous and arterial thromboembolism, cerebral venous thrombosis, pulmonary thromboembolism, acute stroke, capillary leak syndrome, Guillain–Barré syndrome, thrombotic thrombocytopenic purpura, cutaneous rash
4	Whole killed vaccines	BBIBP-CorV, CoronaVac, BBV152	Pain at injection site, fever, fatigue, myalgia, dizziness, arm numbness, headache, ear symptoms, joint pain, itchy rash, pityriasis rosea, pustular psoriasis, nausea, chest pain	Neurological complications, type I Kounis syndrome, reactive arthritis
5	DNA vaccines	ZyCoV-D	Tenderness at injection site, fever, itching, joint pain, diarrhoea	Enteric fever

## Data Availability

Not applicable.

## Abbreviations

ACE2	Angiotensin Converting Enzyme 2
CART	Chimeric Antigen Receptor T cell
CD	Cluster of Differentiation
CI	Confidence Interval
CoV	Corona Virus
GVHD	Graft Versus Host Disease
HCT	Haematopoietic Cell Transplantaion
IFN	Interferon
Ig	Immunoglobulin
IL	Interleukin
LRT	Lower respiratory tract
MERS	Middel-East Respiratory Syndrome
MHC	Major Histocompatibility Complex
NK	Natural Killer
PRNT	Plaque Reduction Neutralization Antibody
RBD	Receptor Binding Domain
SARS	Severe Acute Respiratory Syndrome
STL	Serum Mast Cell Tryptase Level
Th1	T helper cell type 1
TLR	Toll Like Receptor
TNF-α	Tumor Necrosis Factor α
